# High-resolution mapping of metal ions reveals principles of surface layer assembly in *Caulobacter crescentus* cells

**DOI:** 10.1016/j.str.2021.10.012

**Published:** 2022-02-03

**Authors:** Matthew Herdman, Andriko von Kügelgen, Danguole Kureisaite-Ciziene, Ramona Duman, Kamel El Omari, Elspeth F. Garman, Andreas Kjaer, Dimitrios Kolokouris, Jan Löwe, Armin Wagner, Phillip J. Stansfeld, Tanmay A.M. Bharat

**Affiliations:** 1 Sir William Dunn School of Pathology, University of Oxford, Oxford OX1 3RE, UK; 2 Structural Studies Division, MRC Laboratory of Molecular Biology, Cambridge CB2 0QH, UK; 3 Diamond Light Source, Harwell Science & Innovation Campus, Didcot OX11 0DE, UK; 4 Department of Biochemistry, University of Oxford, Oxford OX1 3QU, UK; 5 School of Life Sciences and Department of Chemistry, Gibbet Hill Campus, University of Warwick, Coventry CV4 7AL, UK

**Keywords:** S-layer, bacteria, *Caulobacter crescentus*, metal-ion-binding proteins, fluorescence microscopy, cryo-EM, cryo-ET, long-wavelength X-ray diffraction

## Abstract

Surface layers (S-layers) are proteinaceous crystalline coats that constitute the outermost component of most prokaryotic cell envelopes. In this study, we have investigated the role of metal ions in the formation of the *Caulobacter crescentus* S-layer using high-resolution structural and cell biology techniques, as well as molecular simulations. Utilizing optical microscopy of fluorescently tagged S-layers, we show that calcium ions facilitate S-layer lattice formation and cell-surface binding. We report all-atom molecular dynamics simulations of the S-layer lattice, revealing the importance of bound metal ions. Finally, using electron cryomicroscopy and long-wavelength X-ray diffraction experiments, we mapped the positions of metal ions in the S-layer at near-atomic resolution, supporting our insights from the cellular and simulations data. Our findings contribute to the understanding of how *C. crescentus* cells form a regularly arranged S-layer on their surface, with implications on fundamental S-layer biology and the synthetic biology of self-assembling biomaterials.

## Highlights

• Live imaging shows Ca^2+^-dependent expansion of the *C. crescentus* S-layer
• Molecular simulations reveal Ca^2+^-binding properties of the S-layer
• Ca^2+^ ion mapping in three-dimensional crystals using in-vacuum X-ray anomalous diffraction
• Ca^2+^ replacement by Ho^3+^ allows cryo-EM mapping of heavy metals

## Graphical abstract

**Figure:**
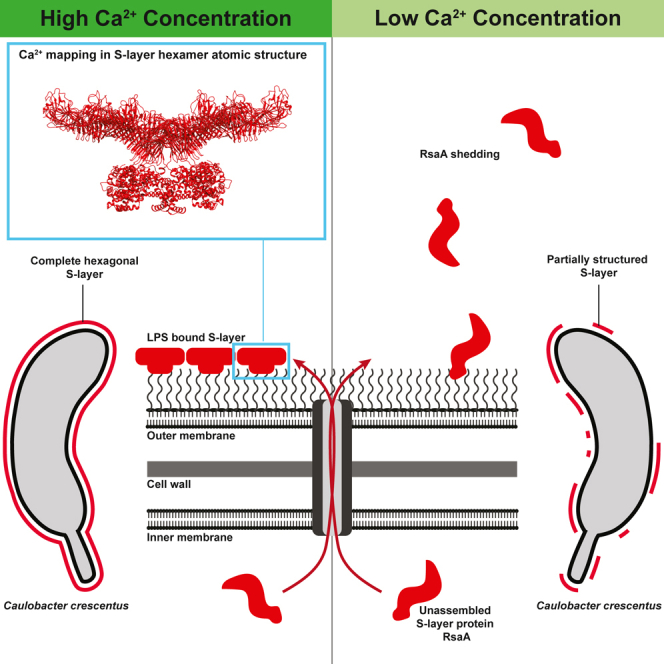

## Teaser

*Caulobacter crescentus* is a model used to study prokaryotic S-layer assembly. Herdman et al. use live-cell imaging, cryo-EM, long-wavelength X-ray diffraction, and molecular simulations to study metal-ion-dependent S-layer formation. They demonstrate the essential role of Ca^2+^ ions in the stabilization, oligomerization, and assembly of the S-layer on cells.

## Introduction

Envelopes are the key platform for cellular interactions with the environment, critical for the regulation of import and export of materials, motility, and the cellular adherence to surfaces. While cell envelopes of prokaryotes are chemically and structurally diverse, many bacterial and almost all archaeal cells are encompassed by a paracrystalline, proteinaceous, macromolecular sheath known as a surface layer (S-layer) (Sara and Sleytr, 2000; Bharat et al., 2021; Sleytr and Beveridge, 1999; Beveridge, 1994; Messner and Sleytr, 1992; Fagan and Fairweather, 2014). S-layers are two-dimensional lattices formed by oligomerization of their constituent S-layer proteins (SLPs), which are often the most abundant proteins in prokaryotic cells (Fagan and Fairweather, 2014; Sara and Sleytr, 2000; Rachel et al., 1997; Engelhardt, 2007a; Engelhardt and Peters, 1998; Lupas et al., 1994). This means that SLPs are also among the most abundant proteins found in nature (Pum et al., 2013).

As S-layers are the outermost component in many prokaryotic cells, they are implicated in many aspects of cellular physiology, including determination and maintenance of cell shape, protection from biomineralization, protection from predators, and evasion of the immune system during host infection (Sleytr et al., 2014; Sara and Sleytr, 2000). Despite a high level of sequence diversity in SLPs, they appear to share several features at the structural and functional levels (Fagan and Fairweather, 2014; Bharat et al., 2021; Engelhardt and Peters, 1998). However, it is currently not clear how similar the different SLPs are in terms of their fold. Advances in structural biology and imaging techniques (Beck and Baumeister, 2016; Oikonomou and Jensen, 2017) have improved our understanding of S-layer biogenesis and assembly, revealing that S-layers have primarily a bipartite arrangement, often with separated lattice-forming and cell-anchoring domains in their constituent SLPs (Bharat et al., 2021; von Kügelgen et al., 2020; Gambelli et al., 2019; Phipps, 1990; Baumeister and Lembcke, 1992; Wildhaber and Baumeister, 1987; Pum and Sleytr, 2014; Veith et al., 2009). Another common feature of S-layers is lattice assembly mediated by the presence of divalent metal ions in the extracellular environment (Engelhardt, 2007a, 2007b), which has been observed in Archaea (Cohen et al., 1991; Kessel et al., 1988), Gram-positive (Baranova et al., 2012; Lupas et al., 1994), and Gram-negative bacteria (von Kügelgen et al., 2020; Bharat et al., 2017; Herrmann et al., 2020).

**Figure 1:**
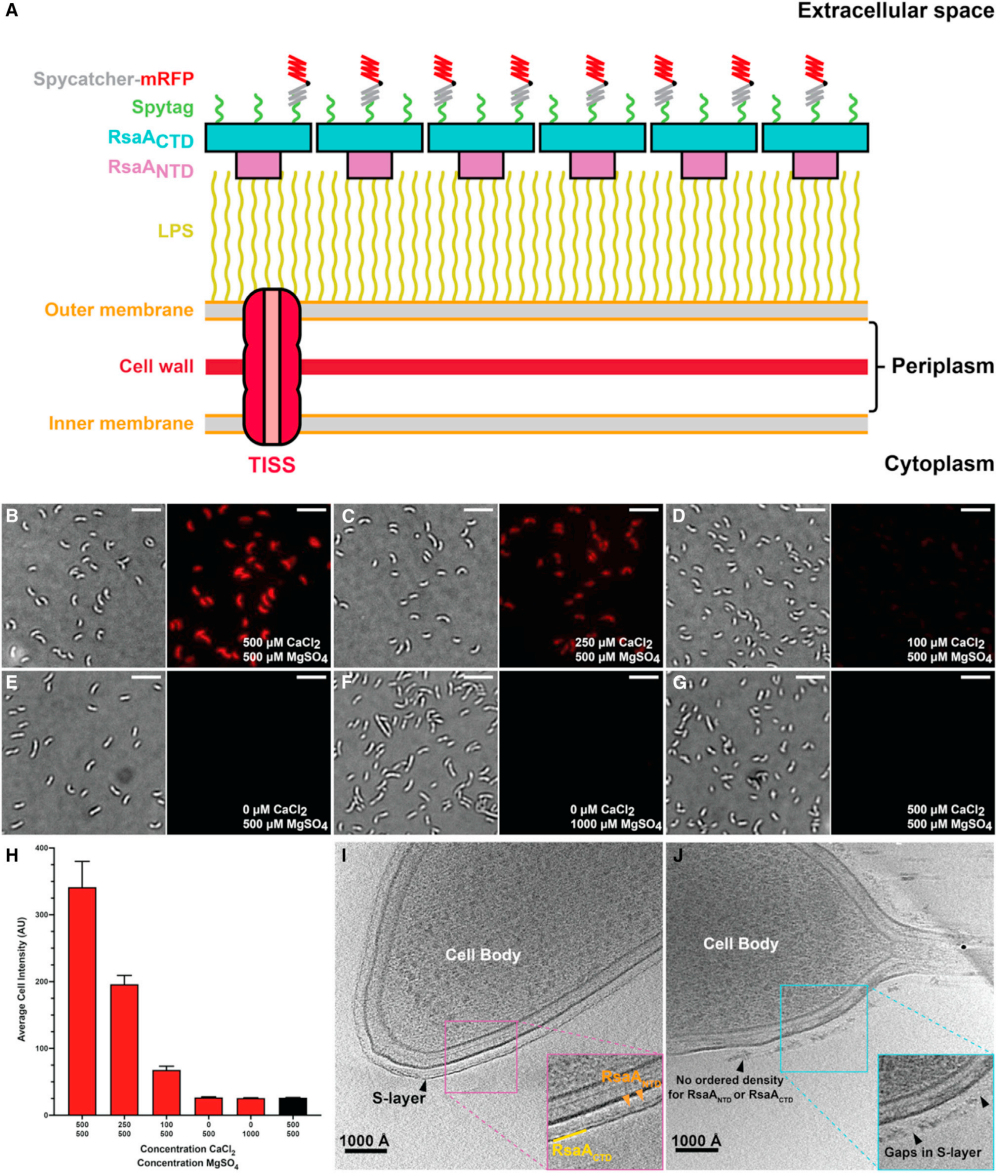
Ca^2+^ ions are critical for S-layer retention on the *C. crescentus* cell surface (A) Cartoon representation of the *C. crescentus* cell surface and the fluorescent tagging approach used in this study. (B–F) *C. crescentus* cells expressing RsaA-467-SpyTag were grown to mid-log phase in M2G medium containing (B) 500 μM CaCl_2_, (C) 250 μM CaCl_2_, (D) 100 μM CaCl_2_, (E) no additional CaCl_2_, (F) 1,000 μM MgSO_4_ and no additional CaCl_2_ and incubated with SpyCatcher-mRFP1. (G) A control sample of cells grown in the same conditions as (B), but without SpyCatcher-mRFP1 labeling. Cellular localization and intensity of the SpyCatcher-mRFP1 signal is strongest in cells grown in medium with 500 μM CaCl_2_. SpyCatcher-mRFP1 signal is absent in cells grown in M2G medium with no added CaCl_2_ (E) and (F) and in the control sample (G). Brightfield (left) and RFP (right) channel contrasts have been normalized across each image. Scale bars: 10 μm. (H) Average cell intensity was quantified using the MicrobeJ plugin for ImageJ/Fiji (Schindelin et al., 2012). Each column represents the average fluorescence intensity of at least 50 cells labeled using the same experimental setup, normalized for cell size. The bar representing unlabeled control cells is shown in black. The graph shows a reduction of SpyCatcher-mRFP1 signal correlated with the reduction of available CaCl_2_ in the medium. Error bars denote standard deviation. (I and J) Six-nanometer slices through reconstructed tomograms of *C. crescentus* CB15N grown in M2G medium with (I) 500 μM CaCl_2_ or (J) 100 μM CaCl_2_. Cells grown in 500 μM Ca^2+^ have a fully assembled S-layer encompassing the entire cell (the continuous outer S-layer lattice and discrete inner domains are marked), as seen in previous studies and consistent with the observations from our fluorescence imaging data. Cells grown in minimal medium with 100 μM Ca^2+^ produce an incomplete S-layer lacking a regular structure that remains associated with the underlying LPS (clear gaps in the S-layer are marked). See also Figure S1.

In cells of the bacterial species *Caulobacter crescentus* (also known as *Caulobacter vibrioides)*, the S-layer is composed of a single 1,026-amino acid residue SLP called RsaA (Smit et al., 1992). RsaA has a canonical bipartite arrangement (Figure 1A), with a lattice-forming C-terminal domain (RsaA_CTD_), consisting of residues 251–1,026, and a cell-anchoring N-terminal domain (RsaA_NTD_), consisting of residues 1–250. The assembly of the RsaA S-layer is divalent metal-ion dependent (Nomellini et al., 1997; Bharat et al., 2017; Herrmann et al., 2020), as, without divalent metal ions, S-layer formation is inhibited both *in vitro* and on cells (Bharat et al., 2017). Furthermore, cryoelectron microscopy (cryo-EM) and cryoelectron tomography (cryo-ET) investigations have demonstrated that binding of RsaA to lipopolysaccharide (LPS) molecules on the *C. crescentus* cell surface is also regulated by Ca^2+^ ions (von Kügelgen et al., 2020). In both the X-ray structure of RsaA_CTD_ (Bharat et al., 2017) and the cryo-EM structure of RsaA_NTD_ bound to LPS (von Kügelgen et al., 2020), the presence of several functional positively charged divalent ions were proposed. However, the identity of these metal ions, and their role in S-layer assembly, could not be determined with much confidence. In this study, we have used fluorescent tagging and optical microscopy to demonstrate the importance of Ca^2+^ ions for the assembly of functional S-layers on *C. crescentus* cells. To understand these cellular observations at the molecular level, we utilized all-atom molecular dynamics (MD) simulations of the RsaA S-layer bound to LPS, where we probed the binding of Ca^2+^ and other ions to the S-layer. Experimentally, metals were unambiguously identified using the particle-induced X-ray emission (PIXE) technique. Using cryo-EM structure determination of a Holmium (Ho^3+^) and LPS-bound RsaA_NTD_, and long-wavelength X-ray diffraction studies on RsaA_CTD_ assembled with Ca^2+^ into stacked sheets in three-dimensional crystals, we report the positions and identities of almost all metal ions in the S-layer, describing 108 experimentally confirmed Ca^2+^ ions bound to each RsaA hexamer in the S-layer. This study allows us to understand metal-ion-dependent S-layer assembly at the atomic level, demonstrating how the cell envelope of *C. crescentus* is built by the binding of many Ca^2+^ ions to the S-layer. We further show at the molecular level why Ca^2+^ binding is functionally important for both lattice assembly and LPS binding for anchoring in the cell envelope.

## Results

### Ca^2+^ ions are critical for the retention and display of the *Caulobacter crescentus* S-layer on cells

To investigate the effect of Ca^2+^ ion concentration on the formation of the S-layer on cells, we employed a strain of *C. crescentus* (CB15N Δ*sapA rsaA467*:SpyTag) that endogenously expresses a single version of RsaA in which a 45-amino acid residue SpyTag peptide has been added at position 467, in an extracellular-oriented region in the RsaA_CTD_ (Figure 1A). This peptide allows irreversible labeling of the cell surface with another polypeptide that contains the SpyCatcher protein (Charrier et al., 2019; Reddington and Howarth, 2015). These cells were grown to mid-log phase in M2G defined medium (without additional Ca^2+^) (Johnson and Ely, 1977), supplemented with differing amounts of CaCl_2_ (see STAR Methods). Cells were grown in the presence of SpyCatcher-mRFP1 (10 μM) to achieve the maximum labeling of SpyTag peptides on the S-layer that is sterically possible, representing ∼25% of cell-surface-bound RsaA (Charrier et al., 2019). *C. crescentus* cells grew poorly in medium containing low concentrations of Ca^2+^ ions, and, in M2G medium depleted of CaCl_2_, extended lag phases as long as 36 h were observed.

We observed that cells grown in M2G medium with normal levels of CaCl_2_ (500 μM) (Ely, 1991) showed an intense fluorescent signal (Figure 1B), indicating that cells were expressing and displaying an S-layer with a SpyTag, which could be readily labeled using SpyCatcher-mRFP1, in line with previous results on wild-type and mutant *C. crescentus* cells (Comerci et al., 2019; Charrier et al., 2019). At slightly lowered concentrations of CaCl_2_ (250 μM), cells displayed a fluorescent signal (Figure 1C); however, quantification showed that the average intensity value for each cell (corrected for cell size) was almost half that of cells grown in 500 μM CaCl_2_ (Figure 1H). An even larger effect was observed when the Ca^2+^ ions in the medium were decreased further (100 μM; Figure 1D); the fluorescent signal in cells was greatly diminished, but detectable on averaging fluorescence values from multiple cells. When no additional CaCl_2_ was added to the medium (Figure 1E), the resulting cell fluorescence readings were indistinguishable from unlabeled control cells (Figure 1G) and drastically lower than cell fluorescence intensities seen in cells in 500 or 250 μM CaCl_2_. These observations support the hypothesis that the formation of a viable S-layer is strongly dependent on the concentration of Ca^2+^ in the growth medium and shows that even a small reduction in the available CaCl_2_ can significantly affect the retention and display of the S-layer on cells, in agreement with other reports (Herrmann et al., 2020). To probe the Mg^2+^ dependence of S-layer assembly, we included a sample of *C. crescentus* cells grown in M2G containing 0 μM CaCl_2_ and 1,000 μM MgSO_4_ (Figure 1F). Despite a 2-fold increase in Mg^2+^ ions in this specimen, the fluorescence from these cells was similar to cells grown in 0 μM CaCl_2_, and ordinary M2G medium (Figure 1E) and the unlabeled control cells (Figure 1G). This suggests that S-layer biogenesis is specifically Ca^2+^ dependent and that Mg^2+^ is not sufficient as a replacement, in line with previous reports (Bharat et al., 2017).

To better understand the composition and arrangement of the S-layer in reduced (100 μM) and normal (500 μM) Ca^2+^ ion concentrations, we collected cryo-ET data from *C. crescentus* cells grown in medium with differing Ca^2+^ ion concentrations. Reconstructed tomograms from each sample (Figures 1I and 1J) showed that cells grown under normal conditions (500 μM Ca^2+^ ions in the medium) retained and possessed a complete S-layer across the entire cell surface with a clear repeating, hexagonal arrangement (Figures 1I and S1), as expected based on previous EM studies (Nomellini et al., 1997; Smit et al., 1992; Bharat et al., 2017). However, cells grown under decreased (100 μM) Ca^2+^ concentrations displayed only a partial, cell-associated S-layer that was irregular in its arrangement and distorted compared with the optimal sample (Figures 1J and S1). These cryo-ET results are consistent with our fluorescent imaging (Figures 1B–1H) wherein cells grown in 100 μM CaCl_2_ showed markedly decreased SpyCatcher-mRFP1 labeling compared with cells grown in optimal CaCl_2_ concentrations. Despite the irregularity of the S-layer seen under reduced Ca^2+^ conditions (Figure 1J), the distance of this aberrant S-layer from the cell surface is roughly the same as for the normal S-layer (Figure 1I), suggesting that these RsaA molecules are still attached to the tip of the LPS.

### Reduced Ca^2+^ ion concentrations affect newly assembled S-layer

**Figure 2:**
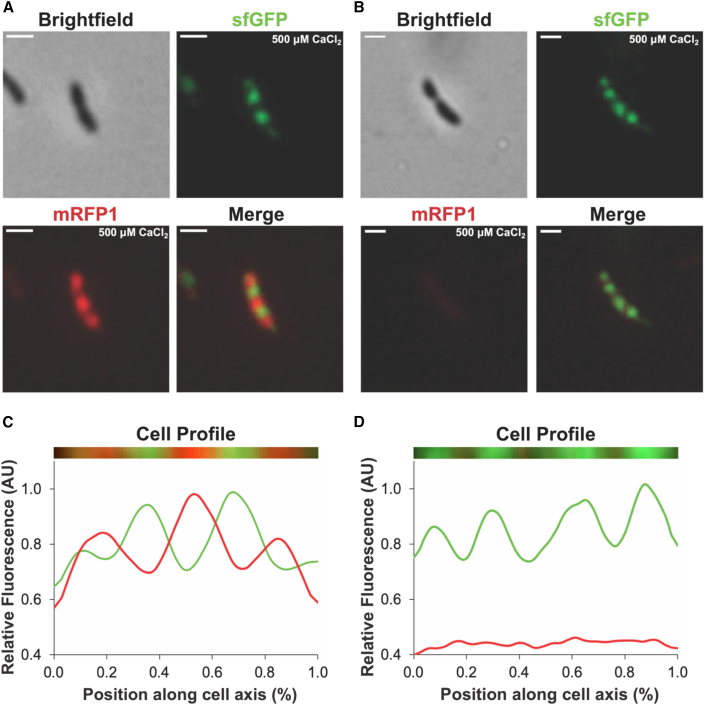
Assembly of new S-layer requires extracellular Ca^2+^ ions *C. crescentus* cells expressing RsaA-467-SpyTag were grown to mid-log phase in M2G medium with 500 μM CaCl_2_ and incubated with SpyCatcher-sfGFP. (A and B) Cells were subsequently incubated with SpyCatcher-mRFP1 in either (A) normal 500 μM CaCl_2_ or (B) minimal 100 μM CaCl_2_. Fluorescence images have been contrasted within their respective channels, and brightfield images are additionally shown for clarity. Intensity thresholds used in all dual-labeling images are further described in Figure S1. Scale bars: 2 μm. (C and D) A representative cell was selected from each labeling condition, shown in (A) and (B), and their fluorescence profiles extracted along the cell axis. The straightened profile of each cell is plotted. Fluorescence intensity (arbitrary units [AU]) is plotted against the position along the cell axis (%) (x and y axes respectively). Cells in both conditions, normal 500 μM (C) and minimal 100 μM CaCl_2_ (D), show a localization of SpyCatcher-sfGFP signal to regions of “old” S-layer along the cell body. However, only in the 500 μM CaCl_2_ treatment (C) is signal for SpyCatcher-mRFP1 relating to “new” S-layer at distinct locations seen, indicating that assembly and expansion of the S-layer in regions of cell growth is dependent on the presence of Ca^2+^.

Recently, an exogenous tagging approach has shown that new *C. crescentus* S-layers are added at defined locations along the cell body (Comerci et al., 2019). We wanted to use this characteristic of S-layer assembly to probe the role of Ca^2+^ ions in S-layer biogenesis using the above SpyTag:SpyCatcher system (Charrier et al., 2019). To this end, we saturated *C. crescentus* cells expressing RsaA-467-SpyTag with SpyCatcher-sfGFP in M2G medium (with normal 500 μM CaCl_2_). After removal of the unbound SpyCatcher-sfGFP, cells were subsequently labeled with SpyCatcher-mRFP1 in the same M2G medium containing either optimal (500 μM) or minimal (100 μM) concentrations of CaCl_2_ (Figure 2).

This sequential, dual-labeling strategy showed regions of old (green-fluorescently tagged) and new (red-fluorescently tagged) S-layer (Figures 2A, 2B, and S1), in line with previous reports using a different labeling strategy (Comerci et al., 2019). Cells grown in M2G medium with 500 μM CaCl_2_ showed localized green fluorescence on the cell body and red fluorescence at mid-cell and at the cell poles (Figure 2A, dividing cell shown). Contrastingly, cells transferred from medium supplemented with 500 μM CaCl_2_ to medium containing 100 μM CaCl_2_ for the second labeling only showed fluorescence in the green channel, as seen in a dividing *C. crescentus* cell with four distinguishable SpyCatcher-sfGFP foci (Figure 2B). We would have expected the gaps between the green foci along the cell, containing the new S-layer, to be labeled red. Therefore, while the labeled old S-layer was associated consistently with the cell body in both Ca^2+^ concentrations, the new S-layer was not observed with fluorescence microscopy in minimal (100 μM) Ca^2+^ concentrations, in line with cryo-ET results shown in Figures 1I and 1J. Fluorescence intensity profiles of cells from the two different conditions were extracted, straightened, and plotted, and showed a clear difference between the normal and minimal Ca^2+^ treatments. The presence of green fluorescence in both labeling conditions confirms that previously secreted (old) S-layer is at least partially retained by cells even when the concentration of CaCl_2_ was greatly reduced. However, the presence of previously polymerized S-layer is in itself insufficient for integration of new RsaA, and additional Ca^2+^ ions are still required for proper assembly of the new S-layer.

### MD simulations of the RsaA S-layer lattice show the location of stable Ca^2+^ ions

Following our studies on the Ca^2+^-dependent formation of the cellular S-layer in *C. crescentus* cells, we wanted to further explore the biochemical and biophysical underpinnings of RsaA binding to Ca^2+^ ions at the atomic (or ionic) level. Based on previous structural biology data, full-length RsaA has been predicted to contain 22 putative Ca^2+^-binding sites; 19 in RsaA_CTD_ (Bharat et al., 2017) (numbered 1–19) and three in RsaA_NTD_ (numbered 20–22) (von Kügelgen et al., 2020). While these sites were all proposed based on unexplained electron or cryo-EM density bound to negatively charged amino acid residue side chains, they have never been directly confirmed to be Ca^2+^-binding sites, and could conceivably correspond to other ions or chemical entities.

**Figure 3:**
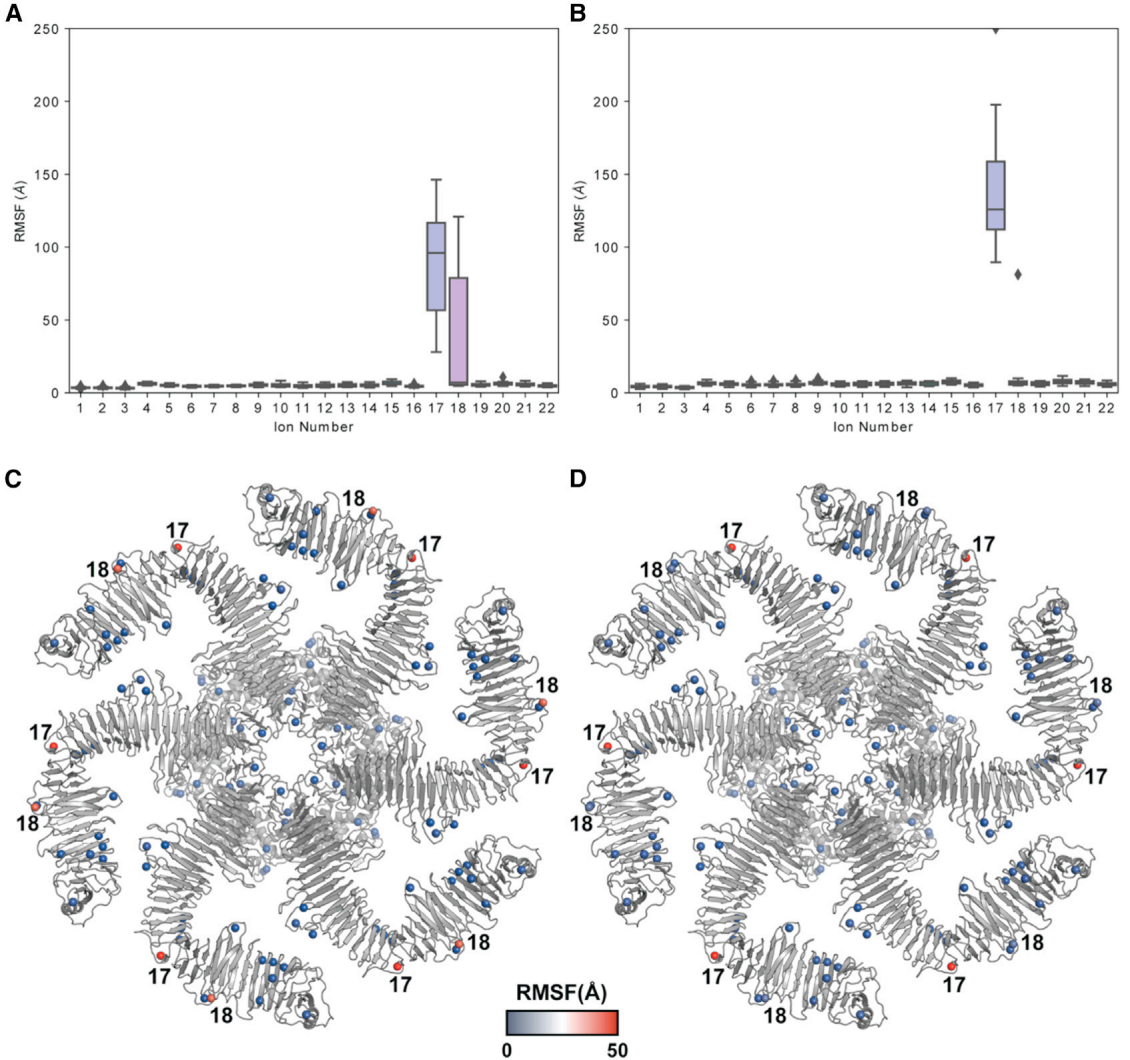
MD simulations of metal-ion binding in the RsaA lattice (A) RMSF values of metal ions in positions 1–22 are shown within our MD simulations performed with all sites occupied by Ca^2+^. All Ca^2+^ ions are stably bound in the RsaA hexagonal sheet except those at positions 17 and 18. (B) When positions 17 and 18 are instead replaced with K^+^ and Mg^2+^ respectively (without any changes to other ion-binding positions), position 18 shows drastically reduced RMSF. (C and D) Ion-binding sites are annotated within the protein ribbon diagram in (C) only Ca^2+^ and (D) containing Ca^2+^, K^+^, and Mg^2+^, with the ions color-coded on a blue-white-red RMSF scale (color calibration bar, bottom).

To approach this problem, we developed an MD simulation framework of a fully solvated, full-length RsaA hexamer. This hexamer was configured into a hexagonal-prism-shaped unit cell, allowing formation of all protein-protein interactions along the plane of the S-layer lattice. These MD simulations also included RsaA_NTD_ bound to the O antigen of LPS (Figure S2). All-atom simulations with all 22 proposed binding sites occupied by Ca^2+^ ions showed that most Ca^2+^ ions remained stably attached to their binding sites in RsaA throughout the 100-ns length of the simulations (Figure 3A). The two notable exceptions were sites 17 and 18 in RsaA_CTD_, which showed large root-mean-square fluctuations (RMSFs), suggesting that those sites were less likely to support Ca^2+^ binding as indicated by the simulation. Based on the geometry of the binding site, and the positively charged ions present in the medium, we performed subsequent simulations where site 17 was replaced with K^+^ and site 18 was replaced with Mg^2+^. These simulations showed that while K^+^ could not bind strongly in site 17, placement of Mg^2+^ in site 18 dramatically reduced the ionic RMSFs in our simulations (Figures 3B and 3D) compared with the case with all sites being occupied with Ca^2+^ (Figures 3A and 3C).

**Figure 4:**
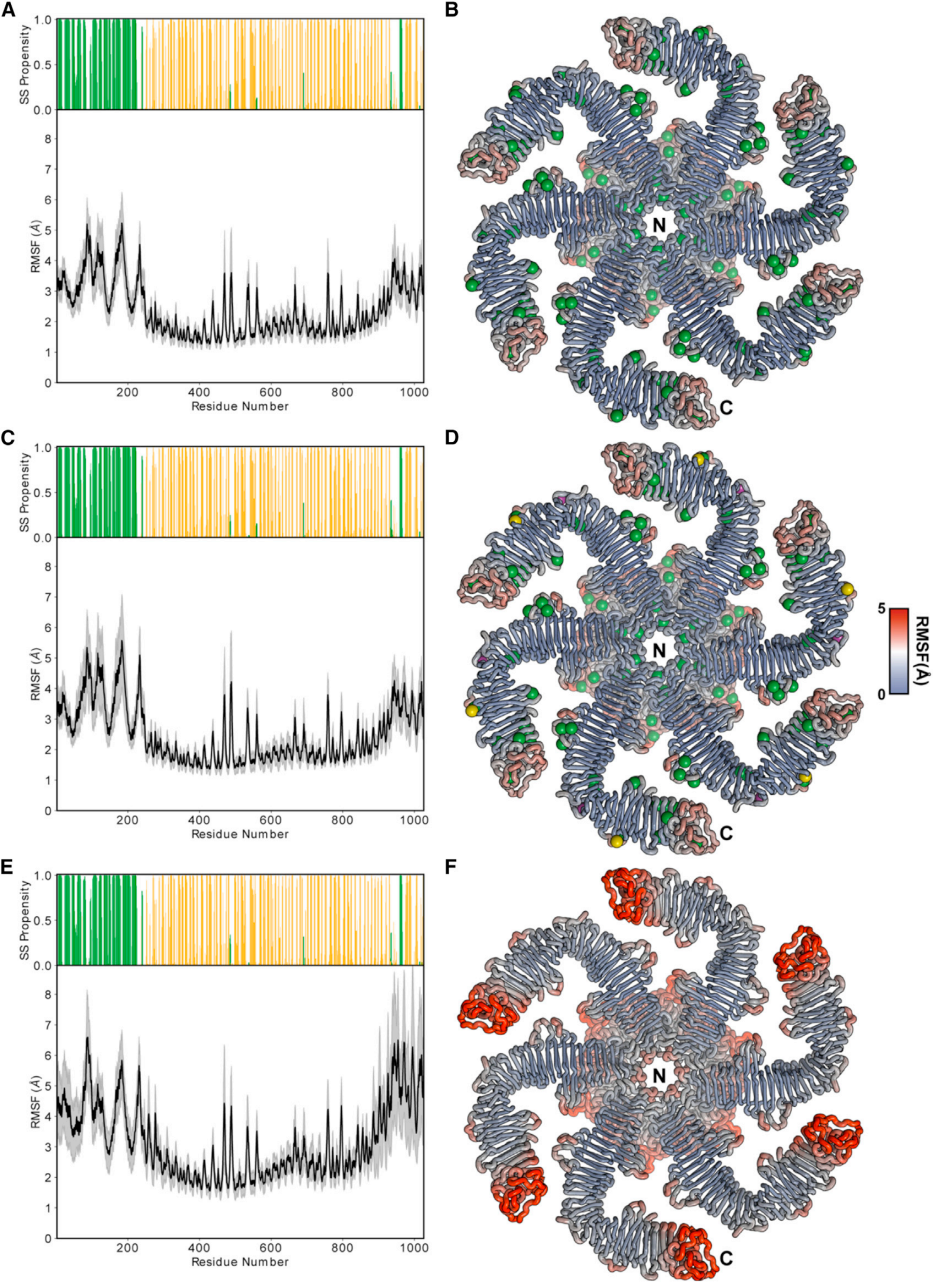
Effect of metal ions on RsaA in MD simulations (A) Secondary structure propensity and RMSF of the backbone carbons of RsaA are shown along the RsaA sequence. Alpha-helical regions are shown in green and beta strands are shown in orange In these simulations, ions 1–22 were all Ca^2+^. (B–D) (B) RMSF plotted onto the RsaA hexamer protein structure color coded using a blue-white-red RMSF scale (color calibration bar on the right applies to B, D, and F), Ca^2+^ shown as green spheres bound at all sites. (C and D) Corresponding figures for simulations conducted with a K^+^ ion replaced in each position 17 (magenta sphere) and an Mg^2+^ ion in every position 18 (gold sphere) bound at site 18, with all other sites bound by Ca^2^^+^. (E and F) Simulations with no ions bound. The greatest fluctuations are observed when no ions are bound, particularly near the C terminus of RsaA (marked “C” near one RsaA subunit ribbon diagram). In all cases, large fluctuations are observed in RsaA_NTD_ (marked “N” near the ribbon diagram), which is not tightly constrained by the protein-protein contacts of the S-layer lattice. See also Figure S3.

In addition to measuring the ionic RMSFs, we also probed the effect of including different metal ions in the RsaA protein within our MD framework. These simulations (Figures 4 and S3) showed that, without Ca^2+^ ions present, RMSF values for protein residues are greatly increased, especially in the C-terminal part of the protein and in the parts involved in protein-protein interface formation that have been proposed previously to depend on bound Ca^2+^ ions (Bharat et al., 2017). This confirmed the expectation from optical microscopy (Figures 1 and 2) that Ca^2+^ binding stabilizes the S-layer lattice, and allowed us to understand our cellular data in the context of the structure of the RsaA lattice. Additionally, the replacement of Ca^2+^ ions at positions 17 and 18 with K^+^ and Mg^2+^ respectively showed similar RMSF to simulations using exclusively Ca^2+^, suggesting the alternative ions are supported within the RsaA lattice to stabilize its structure.

### RsaA binding to Ca^2+^ ions investigated using MicroPIXE and cryo-EM

To further understand our observations made on cells and to verify the predictions made by the MD simulations above, we wanted to directly measure and observe Ca^2+^ binding to RsaA. We first used microbeam particle-induced X-ray emission (MicroPIXE) (Garman and Grime, 2005) to quantify the stoichiometry of calcium bound to purified RsaA by determining the number of calcium atoms relative to the number of sulfur atoms, which are known from the number of methionines and cysteines in the primary sequence. RsaA was purified from cells in a dissociated state using a low-pH treatment, which is not present in the sheet-like oligomeric structure of the S-layer (Bharat et al., 2017), and this purified material was tested for calcium content. MicroPIXE measurements showed that unpolymerized RsaA contained on average about nine Ca atoms (or ions; Figure S4). This experiment confirmed that RsaA is bound to calcium and indicated that the occupation of the other, additional Ca^2+^ ion sites predicted by MD and deduced from the structural work is likely required for lattice formation and sheet assembly.

**Figure 5:**
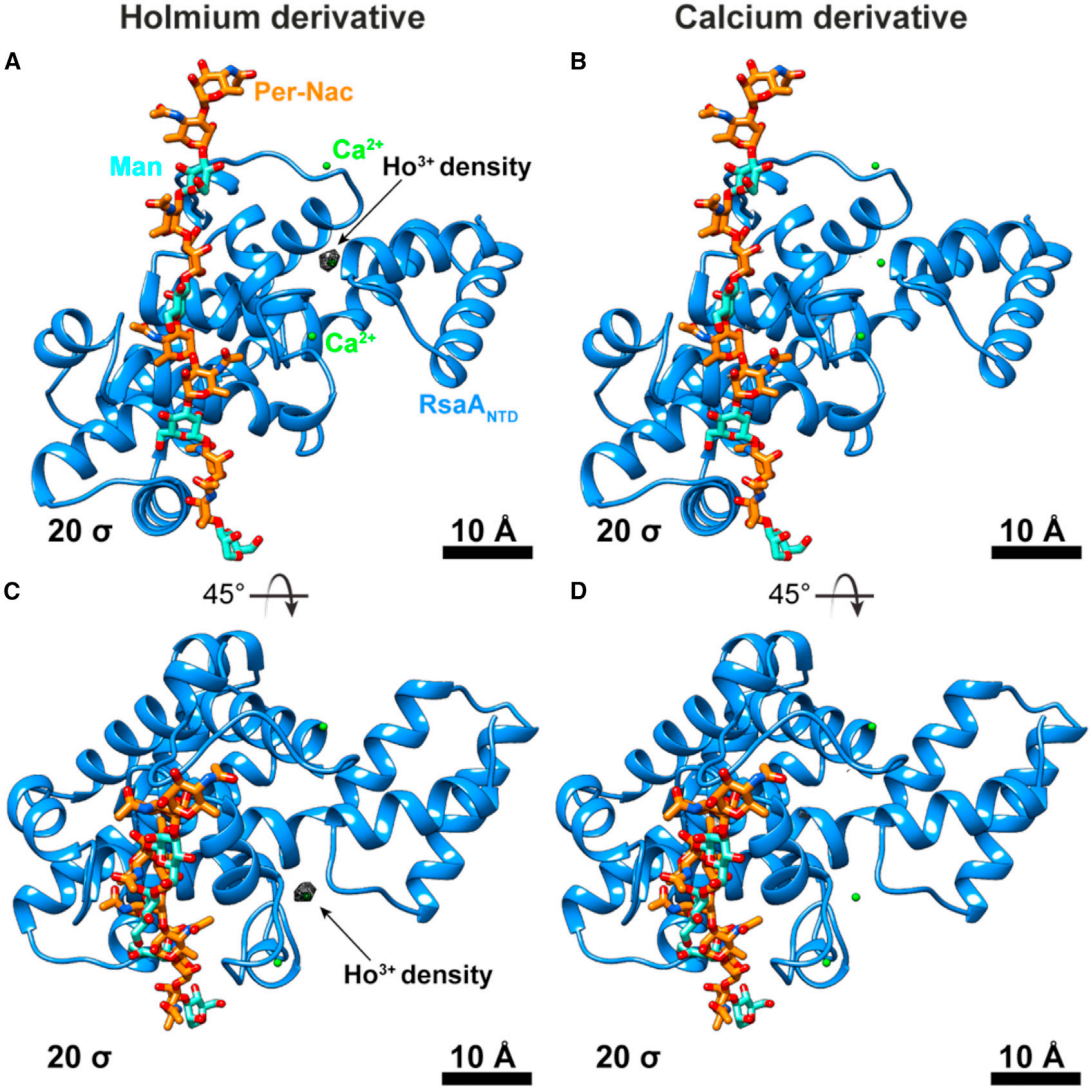
Using cryo-EM of an Ho^3+^ derivative for mapping bound ions to the RsaA_NTD_ Cryo-EM structures of the RsaA_NTD_:PS complex are shown. Atomic models of the RsaA_NTD_ (light blue) shown as ribbon diagram bound to the O-antigen (orange, cyan) of LPS. This complex was proposed to be stabilized by bound Ca^2+^ ions (green spheres). (A) Density map of RsaA_NTD_:PS complex supplemented with 5 mM Ho^3+^ before vitrification shown as black mesh at a contour level of 20 σ. (B) Density map of the control RsaA_NTD_:PS complex at the same contour level. No density is observed at the proposed ion-binding sites. (C and D) Top views of density maps shown in (A) and (B). See also Video S1.

Our next goal was to map the location of Ca^2+^ ions in the RsaA lattice. To verify the Ca^2+^-binding sites in RsaA_NTD_, we reconstituted the RsaA_NTD_:PS (RsaA bound to the O-antigen oligosaccharide of LPS) complex (von Kügelgen et al., 2020) *in vitro*, in the presence of Ho^3+^ ions. Ho^3+^ has a high propensity to replace Ca^2+^ ions (Weis et al., 1991) and we wanted to use this property to spatially confirm the location of Ca^2+^ ions in each RsaA_NTD_ monomer_._ We performed cryo-EM single-particle analysis of the reconstituted complex, producing a 4.3-Å resolution map (Figures 5A and S5; Table S1; Video S1). Comparing this Ho^3+^-bound cryo-EM structure with a Ca^2+^-bound structure solved previously (Figure 5B) (von Kügelgen et al., 2020) showed strong densities in the region of one of the proposed Ca^2+^-binding sites. This experiment shows that at least one of the three proposed Ca^2+^-binding sites in RsaA_NTD_, position 21 (PDB: 6ZYP), is solvent accessible and that the bound Ca^2+^ ion in that site is readily replaced with Ho^3+^ in the complex reconstituted in this study. The proximity between this Ca^2+^ ion and the previously resolved LPS-binding pocket suggests that it might play a role in stabilizing the RsaA_NTD_:PS complex, in line with previous results (von Kügelgen et al., 2020). The other two proposed metal-binding positions in the RsaA_NTD_ were not replaceable by Ho^3+^ under these conditions.

Video S1. Cryo-EM map of the Ho^3+^-bound RsaA_NTD_:PS complex, related to Figure 5 This movie shows different views of the Ho^3+^-bound RsaA_NTD_:PS complex. Due to strong densities corresponding to bound Ho^3+^ ions, the map is contoured at 18 σ away from the mean (black mesh), overlaid on the fitted atomic structure of RsaA_NTD_ and the bound PS.

### Long-wavelength X-ray anomalous diffraction shows Ca^2+^ ion positions in RsaA_CTD_

To experimentally locate Ca^2+^ ions bound to RsaA_CTD_ with atomic precision, we produced RsaA_CTD_ crystals containing S-layer sheets as previously described (Bharat et al., 2017). We used the resulting three-dimensional crystals containing stacked sheets for long-wavelength X-ray anomalous diffraction studies. We utilized the in-vacuum beamline I23 at the Diamond Light Source (Wagner et al., 2016) to allow measurements below and above the X-ray absorption edges of calcium (K edge: 4.0381 keV or 3.0704 Å) and potassium (K edge: 3.6074 keV or 3.4369 Å), energies that are inaccessible to normal beamlines because of strong X-ray absorption and scattering by air. This experiment allowed us not only to identify ions but also to locate their position in the X-ray structure.

**Figure 6:**
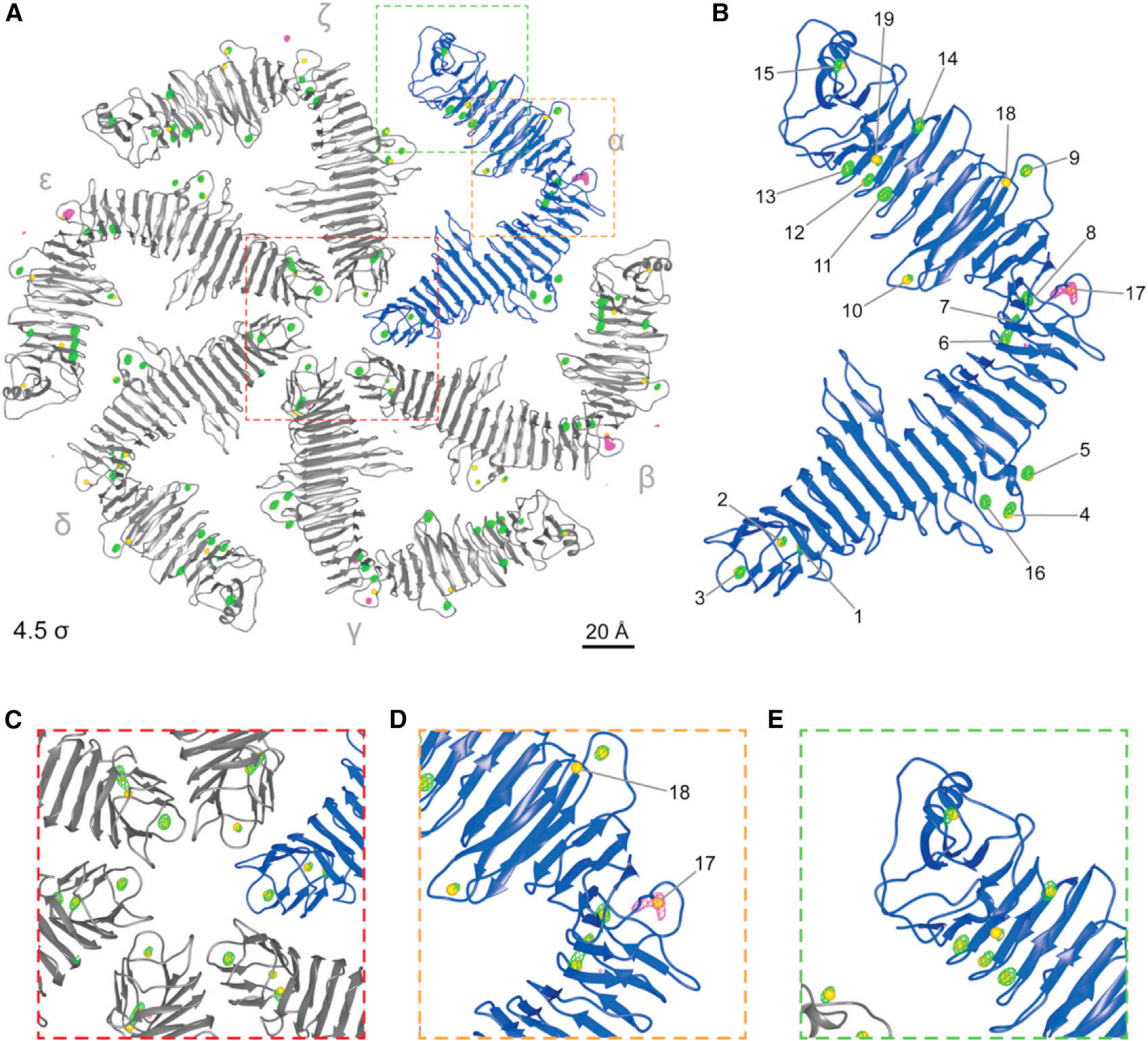
Long-wavelength X-ray diffraction experiments on RsaA_CTD_ crystals (A) The RsaA_CTD_ hexamer (gray and blue) showing the previously proposed metal-ion-binding sites (yellow spheres). Chains are identified as α-ζ with their associated metal ions (see also Figure S6 and Video S2). Densities in anomalous difference maps collected at X-ray energies of 4.1 keV (and absent in 3.95 keV) represent calcium (green mesh). Anomalous difference maps collected at 3.7 keV represent potassium (which were absent in data collected at 3.55 keV, magenta mesh). All maps are displayed at a contour level of 4.5 σ. Positions 1–16 and 19 are consistently coordinated with Ca^2+^ densities (green mesh), with the exception of position 15 and 16 in chain β and position 19 in chain β and ε. Metal-ion-binding site 17 is coordinated with a K^+^ ion in our three-dimensional crystals detected in the dataset collected at an X-ray energy of 3.7 keV in all RsaA monomers except δ. In all monomers, no anomalous density could be observed for position 18. (B) Closeup view of chain α of the RsaA hexamer (blue) showing predicted positions for Ca^2+^ (yellow spheres) and the density from long-wavelength X-ray diffraction experiments (green and magenta mesh) at 4.5 σ. (C–E) Further magnified views of chain α showing RsaA_CTD_ with associated anomalous density. Colored boxes show positions in the full hexamer structure. Central hexameric pore (C), positions 17,18 (D) and the C terminus of RsaA (E).

Anomalous diffraction experiments (Figures 6 and S6; Table S2; Video S2) showed that each RsaA_CTD_ monomer is bound to 17 anomalous scatterers, assigned as Ca^2+^, visible in anomalous difference maps calculated from datasets conducted using X-rays with energies of 4.10 and 3.95 keV. However, not all of the previously proposed positions showed the expected anomalous signal. In line with our MD simulations, positions 17 and 18 showed no evidence of Ca^2+^ binding, verified by the use of different wavelengths in order to distinguish the elements by the characteristic calcium K edge, demonstrating that these sites likely contain other metal ions. In five of the six protein chains present in the RsaA_CTD_ hexamer, position 17 was associated with elongated K^+^ densities in slightly variable locations, as determined using anomalous diffraction performed at 3.70 and 3.55 keV, where no signal from Ca^2+^ is expected. We believe that this site is occupied by K^+^ in the crystals due to the high molarity (70 mM) of KSCN present in the crystallization condition, since this K^+^ ion was not stable in our MD simulations. No associated metal ion was found in position 18 in any of the protein chains, and, based on the MD results, we suggest this to be an Mg^2+^-binding site (Figure 6B), although this prediction cannot be experimentally verified using the wavelengths accessible at the in-vacuum I23 beamline. These long-wavelength anomalous X-ray diffraction experiments allowed us to understand observations made in MD simulations, and helped pinpoint locations of metal ions in the RsaA lattice.

Video S2. Long-wavelength X-ray diffraction experiments on RsaA_CTD_ crystals, related to Figure 6 Anomalous difference maps showing positions of Ca^2+^ ions (green mesh, 4.5 σ) and K^+^ ions (magenta mesh, 4.5 σ) are overlaid on the RsaA_CTD_ atomic structure, as shown in Figure 6.

## Discussion

**Figure 7:**
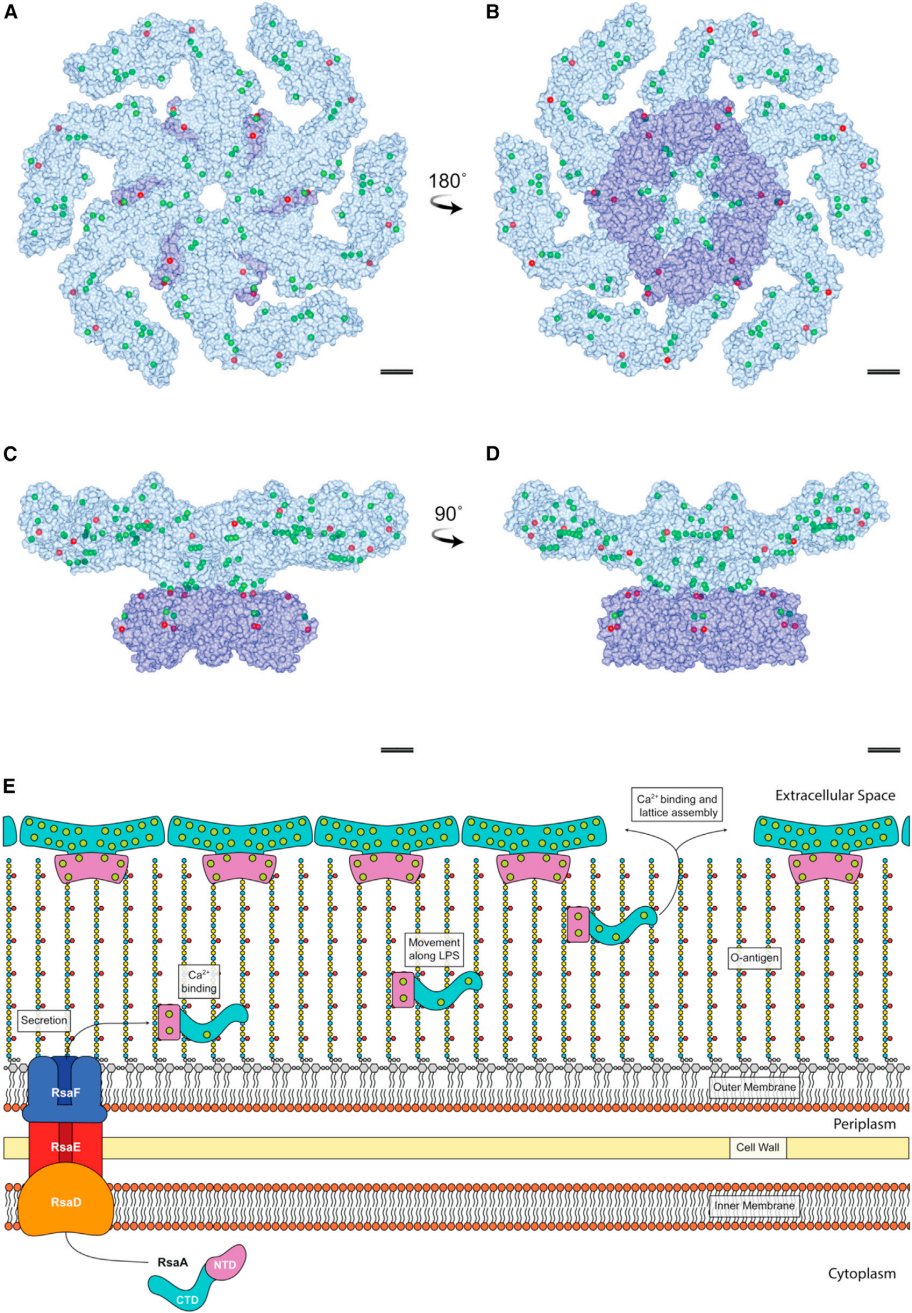
Confirmed identities of metals bound to oligomeric RsaA based on long-wavelength X-ray diffraction and single-particle cryo-EM data. (A and B) Surface model of the RsaA hexamer (RsaA_CTD_ displayed in blue, RsaA_NTD_ in purple) in (A) top view and (B) bottom view with associated metal ions as confirmed by experiments. Confirmed Ca^2+^ ions (positions 1–16, 19, and 21) are shown in green, metal-binding sites with unassigned or no associated ions are displayed in red; i.e., positions 17 (K^+^), 18 (possibly Mg^2+^), and 20 and 22 (probably Ca^2^^+^). (C and D) Orthogonal side views. Scale bar: 50 Å. (E) Schematic showing the secretion of monomeric, unfolded RsaA from the Ca^2+^-limited cytoplasm across the cell envelope by RsaDEF, a type 1 secretion system (ATP-binding cassette [ABC] transporter). Following secretion, RsaA binds Ca^2+^ ions (green circles) and attaches to the O-antigen of the LPS (colored hexagons) via the RsaA_NTD_ (purple), as confirmed by microPIXE and single-particle cryo-EM. RsaA then diffuses along the LPS to the tip of the O-antigen, where the steric hindrance to lattice assembly from the LPS is alleviated, and until it reaches a gap in the growing S-layer. Upon recruiting enough Ca^2+^ ions from the extracellular environment, the RsaA_CTD_ (blue) then oligomerizes and incorporates into the lattice.

Our results provide detailed insights into the metal-ion-binding properties of the surface layer coating *C. crescentus* cells (Figures 7A–7D). Our studies demonstrated that RsaA binds directly to Ca^2+^ ions, and that binding to Ca^2+^ is critical for S-layer biogenesis on cells, as well as for two-dimensional sheet assembly, in line with previous reports (von Kügelgen et al., 2020; Smit et al., 1992; Walker et al., 1994). With our work, we directly and positively confirmed 18 of the 22 proposed Ca^2+^-binding sites (sites 1–16 and 19 in RsaA_CTD_ and site 21 in RsaA_NTD_; Figures 7A–7D). Sites 17 and 18 were confirmed not to bind Ca^2+^, because they could not support Ca^2+^ ion binding in MD simulations, nor were they observed in our long-wavelength X-ray diffraction experiments. Site 21 in RsaA_NTD_ could be replaced with Ho^3+^ and observed with single-particle cryo-EM, suggesting strongly that it is a Ca^2+^-binding site. Since all three proposed Ca^2+^ ion sites (sites 20–22) in RsaA_NTD_ were stable in MD simulations, we expect that these are probably also true Ca^2+^-binding sites, with sites 20 and 22 having less propensity to be replaced by Ho^3+^ under the conditions used, or Ho^3+^ is not capable of substituting for Ca^2+^ in these sites. Our results on mapping Ca^2+^ ions bound to RsaA support our previous proposal that Ca^2+^ ions stabilize the dimeric, trimeric, and hexameric interfaces in the RsaA_CTD_ lattice (Bharat et al., 2017), and demonstrated why the S-layer is heavily dependent on Ca^2+^ ions for sheet assembly.

Despite RsaA_CTD_ and RsaA_NTD_ fulfilling differing and distinct functions, the former mediating crystallization of the outer lattice and the latter anchoring the protein to the O antigen of the LPS, both appear to utilize metal-ion binding to aid their function and assembly. It has been reported that the concentration of Ca^2+^ ions in the cytoplasm of bacteria is tightly regulated (believed to be lower than 100 μM) and markedly lower than in the extracellular environment (Naseem et al., 2008; Holland et al., 1999; Gangola and Rosen, 1987). This fact likely allows RsaA to be expressed in high copy numbers in the cytoplasm in an unfolded or at least unpolymerized state prior to secretion due to the limited availability of free Ca^2+^ ions. Following transport across the cell envelope by its associated type I secretion system (Figure 7E), RsaDEF, RsaA is exposed to the calcium-richer extracellular space where it can bind to Ca^2+^ ions, leading to LPS binding and oligomerization to form the S-layer (Awram and Smit, 1998).

Previous studies have suggested that growth medium with CaCl_2_ concentrations lower than 250 μM trigger shedding of the S-layer into the extracellular milieu and compromise *C. crescentus* fitness and viability (Herrmann et al., 2020). In addition to confirming those results, we further demonstrated that, under these conditions, newly secreted RsaA is inadequately incorporated into the S-layer in *C. crescentus* cells, but that the old S-layer remains at least partially attached to cells at CaCl_2_ concentrations as low as 100 μM. This suggests that RsaA_NTD_ binds more strongly to Ca^2+^ near the surface of *C. crescentus* cells, which in turn leads to strong LPS binding and retention of the S-layer even at lower Ca^2+^ concentrations. At these concentrations, however, the S-layer appears to be aberrant at the ultrastructural level, as observed by cryo-ET, compared with cells grown in normal conditions, failing to form a regularly arranged lattice with repeating symmetrical units (Figures 1 and S1). This indicated that, although RsaA_NTD_ binding to the LPS is not abolished, lattice assembly through RsaA_CTD_ is disrupted. These data together suggested that, while Ca^2+^ in the minimal medium may potentially stabilize recently secreted RsaA and support its retention by the LPS, this low Ca^2+^ concentration is not enough for crystallization of the RsaA_CTD_ into a lattice.

The formation and stabilization of S-layer lattices by metal ions, particularly Ca^2+^ and Mg^2+^, has been observed in multiple species, including other Gram-negative and also Gram-positive bacteria, and archaea (Baranova et al., 2012; Cohen et al., 1991; Kessel et al., 1988; Rodrigues-Oliveira et al., 2019; Berenguer et al., 1988; Farci et al., 2018; Garduno et al., 1995; Engelhardt, 2007a, 2007b). Within this study and in previous attempts to crystallize RsaA (Bharat et al., 2017), we found that Mg^2+^ could not mediate S-layer stabilization or oligomerization. Supplementing M2G medium with an excess of MgSO_4_ to compensate for the exclusion of CaCl_2_ resulted in non-fluorescent cells (Figure 1F), suggesting improper S-layer assembly or S-layer shedding, which is in agreement with previous biochemical reports (Herrmann et al., 2020).

Despite the large variation in SLP sequence and function, metal-ion-dependent assembly is probably a general mechanism to prevent aberrant cytoplasmic assembly of the S-layer in many species, as well as a way to obtain a rigid sheath surrounding cells, assembling with unusually high affinity and co-operativity. In general, metal-ion binding in SLPs is difficult to study, and it is notoriously difficult to measure binding constants in proteins in their cellular context (Yamashita et al., 1990). In this report, we have used an array of complementary methods to study metal-ion dependence of an SLPs at multiple scales from cells to atoms. These methods will be of interest to the structural and cell biology community studying metal-ion-binding proteins and could be applied to other systems where high-resolution information on metal binding is currently unavailable. S-layers have previously been categorized as a form of flexible prokaryotic exoskeleton, influencing cellular shape and stability, with relevance to cell fitness (Engelhardt, 2007a). Additionally, some authors have pointed to S-layers as a frontline defense against biomineralization in the hypersaline environments in which many prokaryotes are found (Chandramohan et al., 2018; Kish et al., 2016). While the metal-ion-binding properties of SLPs have been explored in the context of using S-layers as ion traps and heavy-metal sinks (Park and Taffet, 2019; Pollmann and Matys, 2007; Velasquez and Dussan, 2009; Fahmy et al., 2006), there remains room for exploration of the role of metal ions in S-layer biogenesis.

*C. crescentus* can be regarded as a model organism because of its potential for a variety of synthetic biology applications, in no small part due to its well-ordered and well-characterized S-layer (Nomellini et al., 2007, 2010). Recently, synthetic biology studies have reported the design and use of S-layers for fulfilling multiple functions (Ben-Sasson et al., 2021; Charrier et al., 2019). With more S-layer systems characterized at atomic resolution, such studies will be helped, fueling future research into these fascinating and important two-dimensional protein arrays.

## STAR★Methods

### Key resources table

**Table:** 

REAGENT or RESOURCE	SOURCE	IDENTIFIER
**Bacterial and virus strains**		

*C. crescentus* CB15N	ATCC	ATCC 19089
*C. crescentus* CB15N Δ*sapA rsaA467:*SpyTag	Charrier et al. (2019)	NA
*C. crescentus* CB15N *rsaA*_TEV250_	von Kügelgen et al. (2020)	NA
*E. coli* BL21 (DE3)	ThermoFisher	Cat # EC0114
*E.coli* LMG194	ATCC	ATCC-47090

**Chemicals, peptides, and recombinant proteins**		

10 nm colloidal protein-A gold	CMC Utrecht	PAG 10nm
CaCl_2_	Sigma-Aldrich	Cat# 1023915000
FeSO_4_	Sigma-Aldrich	Cat# 215422
Glucose	Sigma-Aldrich	Cat# D9434
HEPES	Sigma-Aldrich	Cat# G7021
KH_2_PO_4_	Sigma-Aldrich	Cat# P5655
KSCN	Sigma-Aldrich	Cat# P2713
MgSO_4_	Sigma-Aldrich	Cat# M7506
Na_2_HPO_4_	Sigma-Aldrich	Cat# S9763
NH_4_Cl,	Sigma-Aldrich	Cat# A9434
PEG 8000	Sigma-Aldrich	Cat# 89510
SpyCatcher-mRFP1	Charrier et al. (2019)	NA
SpyCatcher003-sfGFP	Keeble et al. (2019)	NA
TAPS	Sigma-Aldrich	Cat# T5316
TEV protease	Produced in-house	NA
Tris (Trizma)	Sigma-Aldrich	Cat# T1503

**Deposited data**		

Composite model of the RsaA S-layer bound to O-antigen of LPS	von Kügelgen et al. (2020)	PDB 6Z7P
RsaA_NTD_ bound to O-antigen of LPS	von Kügelgen et al. (2020)	PDB 6T72
RsaA_CTD_ X-ray structure	Bharat et al. (2017)	PDB 5N8P
Raw cryo-EM data of RsaA_NTD_ bound to Ho^3+^	This manuscript	EMPIAR-10790
Cryo-EM map of RsaA_NTD_ bound to Ho^3+^	This manuscript	EMD-13355
Atomic model of RsaA_NTD_ bound to Ho^3+^	This manuscript	PDB 7PEO
Raw data for X-ray anomalous diffraction studies	This manuscript	Diffraction project datasets I23_AW_RsaA_review https://doi.org/10.18430/M3.IRRMC.5999

**Recombinant DNA**		

pDEST14-SpyCatcher-sfGFP	Keeble et al. (2019)	Addgene Catalogue # 107420
pBAD-SpyCatcher-mRFP1	Charrier et al. (2019)	NA

**Software and algorithms**		

ANODE	Thorn and Sheldrick (2011)	https://shelx.uni-goettingen.de/
CCP-EM suite	Burnley et al. (2017)	https://www.ccpem.ac.uk/
CCP4	Collaborative Computational Project, Number 4, 1994	https://www.ccp4.ac.uk/
ChimeraX	Goddard et al. (2018)	https://www.rbvi.ucsf.edu/chimerax/
Coot 0.8.9.1	Emsley and Cowtan (2004)	https://www2.mrc-lmb.cam.ac.uk/personal/pemsley/coot/
CTFFIND4	Rohou and Grigorieff (2015)	https://grigoriefflab.umassmed.edu/ctf_estimation_ctffind_ctftilt
Excel 16	Microsoft	https://www.office.com
Gromacs 2019	Pronk et al. (2013)	https://manual.gromacs.org/documentation/2019/index.html
GUPIXWIN	Maxwell et al. (1995)	https://www.physics.uoguelph.ca/about-gupix-and-gupixwin
ImageJ/Fiji	Schindelin et al. (2012)	https://www.fiji.sc/
IMOD	Mastronarde and Held (2017)	https://bio3d.colorado.edu/imod/
MicrobeJ (plugin for ImageJ)	Ducret et al (2016)	https://www.microbej.com/
MotionCor2	Zheng et al (2017)	https://emcore.ucsf.edu/
PHENIX	Adams et al (2011)	http://www.phenix-online.org/
Prism 9	GraphPad	https://www.graphpad.com/
Python	https://www.python.org/	https://www.python.org/downloads/
OMDAQ-3	Oxford Microbeams Ltd	http://www.microbeams.co.uk/
Refmac5	Murshudov et al (2011)	https://www.ccp4.ac.uk/
Relion 3.0	Zivanov et al., 2018	https://relion.readthedocs.io/en/release-3.1/Installation.html
SerialEM	Mastronarde (2005)	https://bio3d.colorado.edu/SerialEM/
Tomo3D	Fernandez et al. (2018), Agulleiro and Fernandez (2015)	https://sites.google.com/site/3demimageprocessing/tomo3d
UCSF Chimera	Pettersen et al. (2004)	https://www.cgl.ucsf.edu/chimera/
XDS	Kabsch (2010)	https://xds.mr.mpg.de/
XSCALE	Kabsch (2010)	https://xds.mr.mpg.de/

**Other**		

Quantifoil R2/1.3 holey carbon grids	Quantifoil	NA
R2/2 Cu/Rh 200	Quantifoil	NA
5 mL HisTrap HP column	Sigma-Aldrich	GE29-0510-21
HiLoad Superdex S200 16/600	Sigma-Aldrich	GE28-9893-35
5 mL HiTrap SP HP column	Sigma-Aldrich	GE17-5157-01
5 mL HiTrap Q HP column	Sigma-Aldrich	GE17-1154-01

### Resource availability

#### Lead contact

Further requests should be directed to and will be fulfilled by the lead contact, Dr Tanmay Bharat (tanmay.bharat@path.ox.ac.uk).

#### Materials availibility

This study did not generate new unique reagents.

### Experimental model and subject details

#### Cell lines

Engineered strains of *C. crescentus* CB15N Δ*sapA* expressing RsaA-467-SpyTag (CB15N Δ*sapA rsaA467:*SpyTag) were provided by Prof. Caroline Ajo-Franklin, and grown on PYE agar at 30°C without antibiotics, as reported in Charrier et al. (2019). *E. coli* BL21 (DE3) (ThermoFisher) and LMG194 (ATCC 47090) were grown on LB agar at 37°C with 100 μg/mL ampicillin following transformation. *C. crescentus* and *E. coli* strains were preserved for long term at −80°C as 10% DMSO or 20% glycerol stocks respectively.

### Method details

#### SpyCatcher purification

His-tagged SpyCatcher conjugates were purified as previously described using nickel-affinity chromatography (Charrier et al., 2019). Plasmids pDEST14-SpyCatcher-sfGFP (Keeble et al., 2019) and pBAD-SpyCatcher-mRFP1 were transformed into chemical competent cells *E. coli* BL21 (DE3) and LMG194 cells respectively, and grown on LB agar with 100 μg/mL Ampicillin (LB-Amp). A single colony of each strain was inoculated into 6 L of LB-Amp media and incubated at 37°C with shaking until cells had reached mid-log growth phase. Cells were induced with 0.2% (w/v) arabinose (LMG194) or 0.4 mM Isopropyl β-D-1-thiogalactopyranoside (IPTG) (BL21) and incubated at 20°C for 16 h. Induced cultures were harvested by centrifugation, resuspended in lysis buffer (30 mM Tris/HCl pH 8.0, 500 mM NaCl, 1 mM MgCl_2_, 50 μg/mL DNase, 300 μg/mL lysozyme, and 1x cOmplete Protease Inhibitor), and lysed by five passes through the homogeniser at 15,000 psi (pounds per square inch) pressure. Cell debris were pelleted and the supernatant filtered using a 0.22 μm syringe filter. SpyCatcher proteins were then bound to a 5 mL HisTrap HP column (GE Healthcare) using an ÄKTA pure 25 M system (GE Healthcare) and eluted against the same buffer including 500 mM imidazole over 10 column volumes. Eluates were dialysed overnight with 1:100 (w/w) His_6_-TEV protease at 4°C against 2 L of MilliQ H_2_O. The dialysates were further purified via size exclusion chromatography using a HiLoad Superdex S200 16/600 (prep grade) column; final proteins were eluted in HEPES buffer (25 mM HEPES/NaOH pH 7.5, 150 mM NaCl), and flash frozen in liquid nitrogen and stored at −80°C.

#### RsaA purification by low pH exposure

Wild-type RsaA and RsaA_NTD_ protein was purified as described previously (von Kügelgen et al., 2020; Bharat et al., 2017). In particular, for full-length RsaA, *C. crescentus* CB15N (NA1000) cells were grown in PYE (Poindexter, 1964) medium for 24 h at 25°C with shaking. The resulting culture (4 L) was centrifuged (5000 rcf, 4°C, 30 min) and the pelleted cells were re-suspended in 50 mM HEPES/HCl buffer at pH 2.0 on ice for 10 mins with vigorous shaking. Next, the suspension was centrifuged (16000 rcf, 4°C, 30 min) and the cell pellet was discarded. The pH of the supernatant was adjusted to 7.0 with 5 M NaOH, filtered and loaded onto a 5 mL HiTrap SP HP column (GE Healthcare). The flow-through from the column was collected and dialyzed against 10 mM Tris/HCl pH 8.0 overnight at 4°C. The dialyzed protein solution was loaded onto a 5 mL HiTrap Q HP column (GE Healthcare), washed with 20 mM Tris/HCl pH 8.0 and then eluted with the same buffer containing increasing concentrations of NaCl. Fractions containing pure RsaA were collected and dialyzed against 20 mM Tricine/NaOH pH 8.0 and then concentrated to ∼25 mg/mL. Aliquots were flash frozen in liquid nitrogen and stored at −80°C.

For RsaA_NTD_ protein, cells from the *C. crescentus rsaA*_TEV250_ strain (von Kügelgen et al., 2020), containing the genomic TEV-protease cleavage site, were grown in PYE medium for 24 h at 20°C with shaking at 180 rpm. Six litres of the bacterial culture were centrifuged (5000 rcf, 4°C, 30 min). The pelleted cells were re-suspended in 50 mM HEPES/HCl buffer at pH 2.0 on ice for 10 minutes with vigorous shaking. Next, the suspension was centrifuged (16000 rcf, 4°C, 30 min). The pellet was discarded and the pH of the supernatant was adjusted to 7.0 with 5 M NaOH. The resulting liquid was filtered and loaded onto a 5 mL HiTrap SP HP column (GE Healthcare). The flow-through from the column was collected and dialyzed against 10 mM Tris/HCl pH 8.0 for 3 hours at 4°C. The dialyzed solution was loaded onto a 5 mL HiTrap Q HP column (GE Healthcare), washed with 20 mM Tris/HCl pH 8.0 and then eluted with the same buffer containing increasing concentrations of NaCl. Fractions containing pure RsaA_TEV250_ were collected and cleaved overnight by addition of His_6_-TEV protease in a ratio of 1:100 (w:w). His_6_-TEV protease was removed by loading the protein solution to a 5 mL HisTrap FF column (GE Healthcare). The flow-through was collected, concentrated and loaded to a Superdex S200 16/600 (prep grade) column (GE Healthcare) equilibrated with 25 mM HEPES/NaOH pH 7.5, 100 mM, 1 mM CaCl_2_. RsaA_NTD_ was eluted with the same buffer and fractions containing RsaA_NTD_ were collected and concentrated to 3.7 mg/mL protein concentration (Amicon 10 kDa MWCO). Aliquots were frozen in liquid nitrogen and stored at −80°C.

PS (polysaccharide) was partially released from purified, crude LPS by hydrolysis with acetic acid (1% (v/v), 95°C, 2 hours). The sample was clarified by centrifugation (16000 rcf, 4°C, 30 min) and adjusted to pH 7.0 by addition of 1 M HEPES/NaOH pH 7.0. An excess of purified RsaA_NTD_ was mixed with hydrolysed PS (von Kügelgen et al., 2020) and the mixture was dialyzed against 25 mM HEPES/NaOH pH 7.5, 100 mM NaCl, 1mM MgCl_2_, 1mM CaCl_2_ overnight at 4°C. The sample was loaded to a Superose 6 Increase 10/300 GL column (GE Healthcare) equilibrated with the same buffer. Peak fractions containing oligomeric RsaA_NTD_ were collected, concentrated (Amicon 30 kDa MWCO), flash frozen in liquid nitrogen and stored at −80°C.

#### Light microscopy and image processing

*C. crescentus* CB15N Δ*sapA* expressing RsaA-467-SpyTag (CB15N Δ*sapA rsaA467:*SpyTag) cells were grown in M2G (6.1 mM Na_2_HPO_4_, 3.9 mM KH_2_PO_4_, 9.3 mM NH_4_Cl, 0.5 mM MgSO_4_, 0.01 mM FeSO_4_, 0.2% (w/v) Glucose ) media with a defined concentration of CaCl_2_ (0–500 μM) at 30°C with aeration by shaking to mid-log growth phase. Cells were resuspended to an OD_600_ of 0.1 in the same media supplemented with 10 μM of the appropriate SpyCatcher-FP (fluorescent protein) conjugate. All labelling steps were carried out at 4°C for 16 h, after which point cells were harvested by centrifugation and washed three times in their respective M2G media. Where indicated, cells were resuspended in the appropriate M2G media and incubated at 30°C for 3 h with 10 μM of a contrasting SpyCatcher-FP to promote growth and S-layer labelling. After labelling, cells were harvested, washed as previously and resuspended. All labelling steps were carried out with the specimen protected from light-exposure. 3-5 μL of labelled cell suspensions were spotted onto agarose pads (1% (w/v) agarose in distilled water) enclosed by a 15 mm × 16 mm Gene Frame (ThermoFisher) on a glass slide and sealed with a glass coverslip. Slides were imaged using a 100× objective lens using a Zeiss AxioImager M2 widefield microscope (Carl Zeiss).

Images were background-subtracted and the contrast of each image normalised depending on the fluorescence channel. Using the MicrobeJ plugin for ImageJ (Ducret et al., 2016), cells were outlined and the average pixel intensity value extracted for statistical analysis of the SpyCatcher-FP labelling efficiency. For cell-profile analysis, a 3-pixel line was drawn along the long-axis of the cell through the cell body, straightened, and the pixel values extracted. Pixel intensity was displayed at a relative scale to show localisation of the fluorescence signal along the cell body.

#### Molecular dynamics simulations

Atomistic simulations were run in triplicate for 100 ns using the CHARMM36m forcefield. Simulations were performed at 310 K using the velocity-rescaling temperature coupling algorithm (Bussi and Parrinello, 2007), with a time constant of 0.1 ps and Parrinello-Rahman isotropic pressure coupling of 1 bar with a time constant of 2 ps (Parrinello and Rahman, 1981). Electrostatics were handled using the Particle-Mesh-Ewald method (Darden et al., 1993), and a force-switch modifier was applied to the van der Waals forces. Dispersion corrections were turned off. The parameters for the O-antigen were generated using the CHARMM-GUI (Kim et al., 2017; Lee et al., 2016). All simulations were run using Gromacs 2019 (Pronk et al., 2013). Molecular simulation images and Supplemental Videos of simulations were made in PyMOL. Graphs were plotted using Python and Matplotlib.

#### RsaA crystallisation

Wild-type RsaA protein was crystallised as described previously (Bharat et al., 2017), with RsaA protein solution supplemented with 5 mM CaCl_2_ prior to crystallisation. Initial screens of full-length RsaA were setup using the MRC Laboratory of Molecular Biology’s in-house robotic nano-litre crystallisation facility (Stock et al., 2005). After optimisation, the native RsaA crystals were grown at 19°C by sitting-drop vapour-diffusion in a drop composed of 100 nL of reservoir solution (0.07 M KSCN, 24% (w/v) PEG 8000, 0.075 M TAPS/NaOH pH 8.5) and 100 nL of protein solution at 30 mg/mL. Plate-like crystals appeared in 3–10 days and continued growing to a final size of 300 × 300 × 30 μm^3^. Crystals were flash-cooled with liquid nitrogen for data collection using an additional 25% (v/v) PEG 200 as cryo-protectant.

#### Micro-PIXE

Measurements were carried out at the Ion Beam Centre, University of Surrey, UK (Grime et al., 1991). Characteristic X-ray emission was induced using a 2.0 μm diameter 2.5-MeV proton beam incident on dried protein droplets (volume per droplet approx. 0.1 μL) under vacuum. Emitted X-rays were detected in a high energy resolution solid state lithium drifted silicon detector. Spatial maps were obtained of all elements heavier than magnesium present through scanning the proton beam in x and y over the dried sample, and 3-4 point spectra were collected from each droplet. GUPIX (Maxwell et al., 1995) within OMDAQ-3 (Microbeams Ltd, UK) was used to analyse these spectra to extract the relative amount of each element per protein molecule, particularly calcium, in the sample.

#### Cryo-EM sample preparation

For cryo-EM grid preparation 2.5 μL of purified RsaA_NTD_:PS complex (2.25 mg/mL) was mixed with 5 mM HoCl_3_, incubated on ice for 1.5 h and was then applied to a freshly glow discharged Quantifoil R2/2 Cu/Rh 200 mesh grid, adsorbed for 10 s, blotted for 4 s and plunge-frozen into liquid ethane in a Vitrobot Mark IV (ThermoFisher), while the blotting chamber was maintained at 100% humidity at 10°C. For *C. crescentus* cells, 10 nm protein-A gold (CMC Utrecht) was additionally added to the samples.

#### Cryo-EM data collection

Single-particle cryo-EM data of the Ho^3+^-bound RsaA_NTD_:PS complex were collected on a Titan Krios G3 microscope (ThermoFisher) operating at 300 kV fitted with a Quantum energy filter (slit width 20 eV) and a K2 direct electron detector (Gatan) with a sampling pixel size of 1.08 Å running in counting mode. In total 903 movies with a specimen stage tilt of 0° and 1135 movies with a specimen stage tilt of 30° were collected with a dose rate of 6.578 e^−^/pixel/s on the camera level. The sample was subjected to 8 s of exposure during which a total dose of 44.8 e^−^/Å^2^ was applied, and 20 frames were recorded per movie. For tomographic data collection, the SerialEM software (Mastronarde, 2005) was used as described previously (Sulkowski et al., 2019).

#### Cryo-EM and cryo-ET image processing

For single-particle analysis, movies of the untilted and tilted dataset were motion corrected and dose weighted with MotionCor2 (Zheng et al., 2017) implemented in Relion 3.0 (Zivanov et al., 2018). Contrast transfer functions (CTFs) of the resulting motion corrected micrographs were estimated using CTFFIND4 (Rohou and Grigorieff, 2015). Initial Particles were extracted in a 2x down-sampled 150 pixel × 150 pixel box and classified using reference-free 2D-classification inside Relion 3.0. Particles from classes showing high-resolution features from both datasets were merged, re-extracted in a 300 pixel × 300 pixel box and were subjected to 3D classification using a 30 Å lowpass filtered reference map of EMD-10389 (von Kügelgen et al., 2020). Particles from a class showing clear-separation of the individual RsaA_NTD_ subunits were combined for a focused 3D auto refinement on the central 14 subunits using the output from the 3D classification as a starting model. The final map was obtained from 158,430 particles and post-processed using a soft mask focused on the inner fourteen subunits yielding a resolution of 4.37 Å according to the gold standard Fourier shell correlation criterion of 0.143 (Scheres, 2012) with some anisotropy in Z as judged by directional FSCs (Tan et al., 2017). Cryo-EM single-particle data statistics are summarised in Table S1. Cryo-ET data analysis was performed in IMOD (Mastronarde and Held, 2017) and tomographic reconstruction was carried out using the SIRT algorithm implemented within Tomo3D (Fernandez et al., 2018; Agulleiro and Fernandez, 2015).

#### EM model building and refinement

The atomic coordinates (PDB ID 6T72) of our previous cryo-EM structure (von Kügelgen et al., 2020) of the RsaA_NTD_ oligomer bound to the O-antigen of lipopolysaccharide (LPS) were rigid body fitted into the final post-processed map from Relion 3.0 (Zivanov et al., 2018) using UCSF Chimera (Pettersen et al., 2004). The resulting fitted model was subjected to refinement using Refmac5 (Murshudov et al., 2011) inside the CCP-EM suite (Burnley et al., 2017), as described previously (von Kügelgen et al., 2020). Briefly, reference restraints of the initial structure (PDB ID 6T72) were generated with PROSMART (Nicholls et al., 2014), and these restraints were used in the standard model versus map refinement protocol within Refmac5 (Murshudov et al., 2011). The output refined model was validated using PHENIX (Adams et al., 2011) and the results of the validation are summarised in Table S1.

#### Long wavelength X-ray diffraction

X-ray diffraction data were collected at beamline I23, Diamond Light Source (Wagner et al., 2016), equipped with a Pilatus 12M (Dectris AG, Switzerland) detector, at four energies, 4.1, 3.95, 3.7 and 3.55 keV using the inverse beam method with 20° wedges. Data were processed using XDS (Kabsch, 2010) and half-datasets were merged using XSCALE. Data were collected from several crystals; however, non-isomorphism forbade using multi-crystal averaging to improve the signal and the datasets were treated separately. The structures were solved by molecular replacement using the deposited model (PDB ID 5N8P) with removed metal ions. From the structure factors deposited in the PDB, native omit electron density maps were generated in Phenix (Adams et al., 2011) using the models with removed metal ions. The anomalous difference maps from the long-wavelength data were generated using ANODE (Thorn and Sheldrick, 2011). The positions of anomalous peaks higher than 5.0 σ from datasets both above and below the calcium and potassium K-edge were inspected in COOT (Emsley and Cowtan, 2004) and compared with the difference peaks in the native omit maps. Ca^2+^ and K^+^ ions were modelled in the positions where positive omit difference density peaks (>3.5 σ) overlapped with peaks in the anomalous difference map from data collected above the respective absorption edge while absent in the corresponding map below the edge. Figures illustrating X-ray or cryo-EM structures were prepared using USCF Chimera (Pettersen et al., 2004) or ChimeraX (Goddard et al., 2018). X-ray crystallography data statistics are summarised in Table S2.

### Quantification and statistical analyses

Quantification of micrographs from light microscopy experiments were carried out using MicrobeJ and analysed using GraphPad PRISM, described in detail above. For all figures where appropriate, the number of measurements performed, along with mean and standard deviations are reported within the figures or the figure legends. Estimation of resolution of cryo-EM maps was performed using Fourier Shell Correlation (FSC) analysis.

## Data Availability

The raw cryo-EM data and the final 3D reconstruction of the RsaA_NTD_:PS complex bound to Ho^3+^, along with the fitted atomic model have been deposited in the EMPIAR database (Accession code EMPIAR-10790), the Electron Microscopy Data Bank (Accession code EMD-13355) and the Protein Data Bank (Accession code 7PEO) respectively. Raw data from X-ray anomalous diffraction studies has been deposited at http://www.proteindiffraction.org with the DOI - https://doi.org/10.18430/M3.IRRMC.5999. All these data entries will be released upon publication. This paper does not report original code. Any additional information required to reanalyze the data reported in this paper is available from the lead contact upon reasonable request.

## Supplemental information

Document S1. Figures S1–S6 and Tables S1 and S2

Document S2. Article plus supplemental information
