# Supplementary material for: High-resolution mapping of metal ions reveals principles of surface layer assembly in Caulobacter crescentus cells
Source: Structure. 2022 Feb 3;30(2):215–228.e5. doi: 10.1016/j.str.2021.10.012 (PMC8828063; doi:10.1016/j.str.2021.10.012)
Supplement: Document S1. Figures S1–S6 and Tables S1 and S2 [file mmc1.pdf]

Structure, Volume *30*

Supplemental Information

High-resolution mapping of metal ions reveals principles of surface layer assembly in *Caulobacter crescentus* cells

Matthew Herdman, Andriko von Kügelgen, Danguole Kureisaite-Ciziene, Ramona Duman, Kamel El Omari, Elspeth F. Garman, Andreas Kjaer, Dimitrios Kolokouris, Jan Löwe, Armin Wagner, Phillip J. Stansfeld, and Tanmay A.M. Bharat

Supplementary Information for article titled

High-resolution mapping of metal ions reveals principles of surface layer assembly in *Caulobacter crescentus* cells

Author list

Matthew Herdman[1,#], Andriko von Kügelgen[1,#], Danguole Kureisaite-Ciziene[2], Ramona Duman[3], Kamel El Omari[3], Elspeth F. Garman[4], Andreas Kjaer[4], Dimitrios Kolokouris[4], Jan Löwe[2], Armin Wagner[3], Phillip J. Stansfeld[5,*] and Tanmay A.M. Bharat[1,2,*,Ψ]

Affiliations

[1] Sir William Dunn School of Pathology, University of Oxford, Oxford OX1 3RE, United Kingdom

[2] Structural Studies Division, MRC Laboratory of Molecular Biology, Cambridge CB2 0QH, United Kingdom

[3] Diamond Light Source, Harwell Science & Innovation Campus, Didcot OX11 0DE, United Kingdom

[4] Department of Biochemistry, University of Oxford, Oxford OX1 3QU, United Kingdom

[5] School of Life Sciences and Department of Chemistry, Gibbet Hill Campus, University of Warwick, Coventry CV4 7AL, United Kingdom

[#] These authors contributed equally to this work

***Correspondence to**:

Phillip Stansfeld, email: phillip.stansfeld@warwick.ac.uk

and

Tanmay A.M. Bharat, email: tanmay.bharat@path.ox.ac.uk

[Ψ] **Lead contact**

Other supplementary materials for this manuscript include the following:

Movies S1 to S2

Supplementary Figures

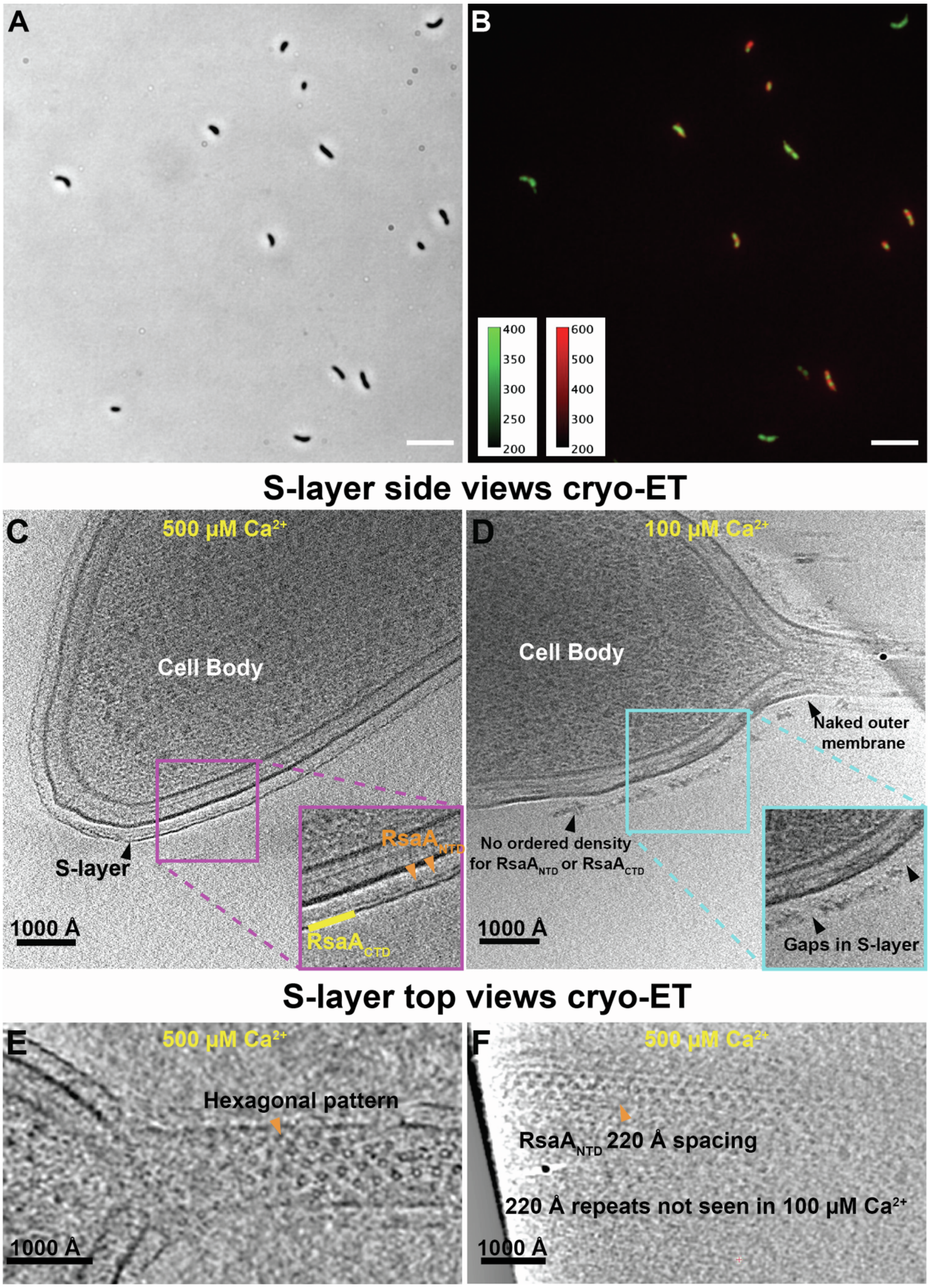

Fig. S1: Fluorescence labelling and cryo-ET of *C. crescentus* cells (related to Fig. 1).

(A) *C. crescentus* cells imaged using brightfield optical microscopy. (B) Composite image from red and green channels showing *C. crescentus* cells sequentially labelled via cell-bound RsaA-467-SpyTag with SpyCatcher-sfGFP (green signal) followed by growth in the presence of SpyCatcher-mRFP1 (red signal). Calibration bars showing the thresholding of the pixel intensity for red and green channels are provided in arbitrary units (AU). Scale bar 10 μm. (C-D) 6 nm slices through reconstructed tomograms of *C. crescentus* CB15N grown in M2G media with (C) 500 μM $CaCl_2$ or (D) 100 μM $CaCl_2$. 500 μM Ca^{2+} results in a fully assembled S-layer encompassing the entire cell (the continuous outer S-layer lattice and discrete inner domains are marked), as seen in previous studies and consistent with the observations from our fluorescence imaging data. Cells grown in minimal 100 μM $CaCl_2$ produce an incomplete S-layer lacking a regular structure that remains associated with the underlying LPS (clear gaps in the S-layer and naked cell membranes are marked). (E-F) Slices (6 nm) through the top of the cell or cell stalk show clear hexagonal S-layer patterns in 500 μM Ca^{2+}, not observed in 100 μM Ca^{2+}.

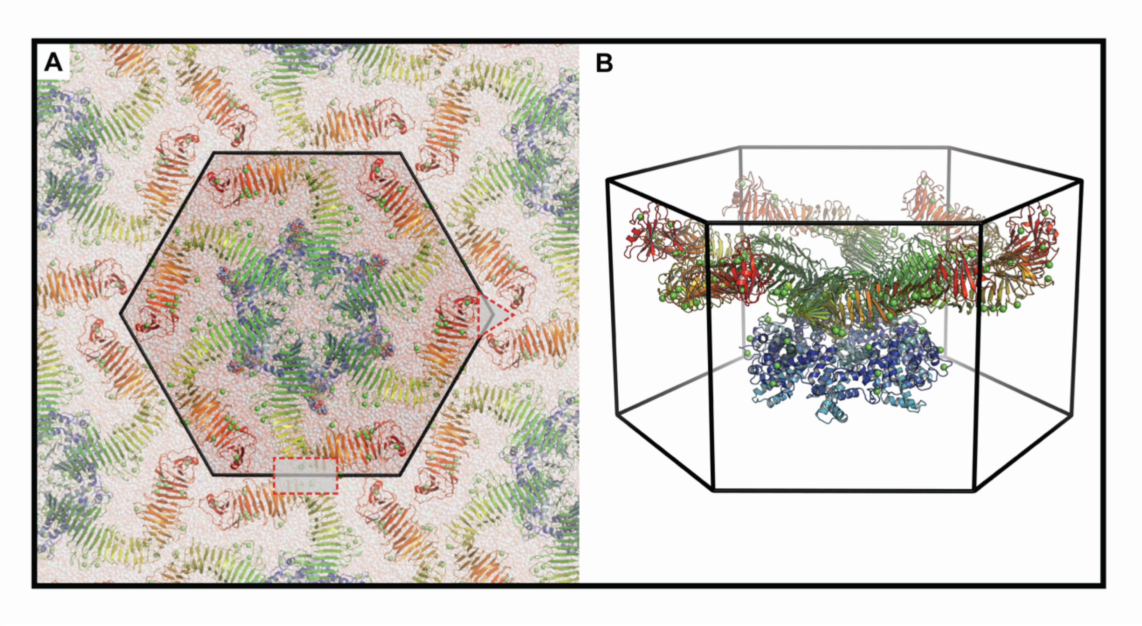

Fig. S2. MD simulations of the complete *C. crescentus* S-layer (related to Fig. 3).

(A) To simulate the complete S-layer of *C. crescentus*, full-length RsaA was placed in a prism-like box, (B) which allowed lattice contacts in a hexagonal arrangement, as found in the cellular S-layer. The RsaA protein was solvated, with multiple metal ions bound, along with the RsaA O-antigen (not shown in the figure above for clarity). This solvated and relaxed lattice was then used in all-atom molecular dynamics simulations to probe metal-ion binding.

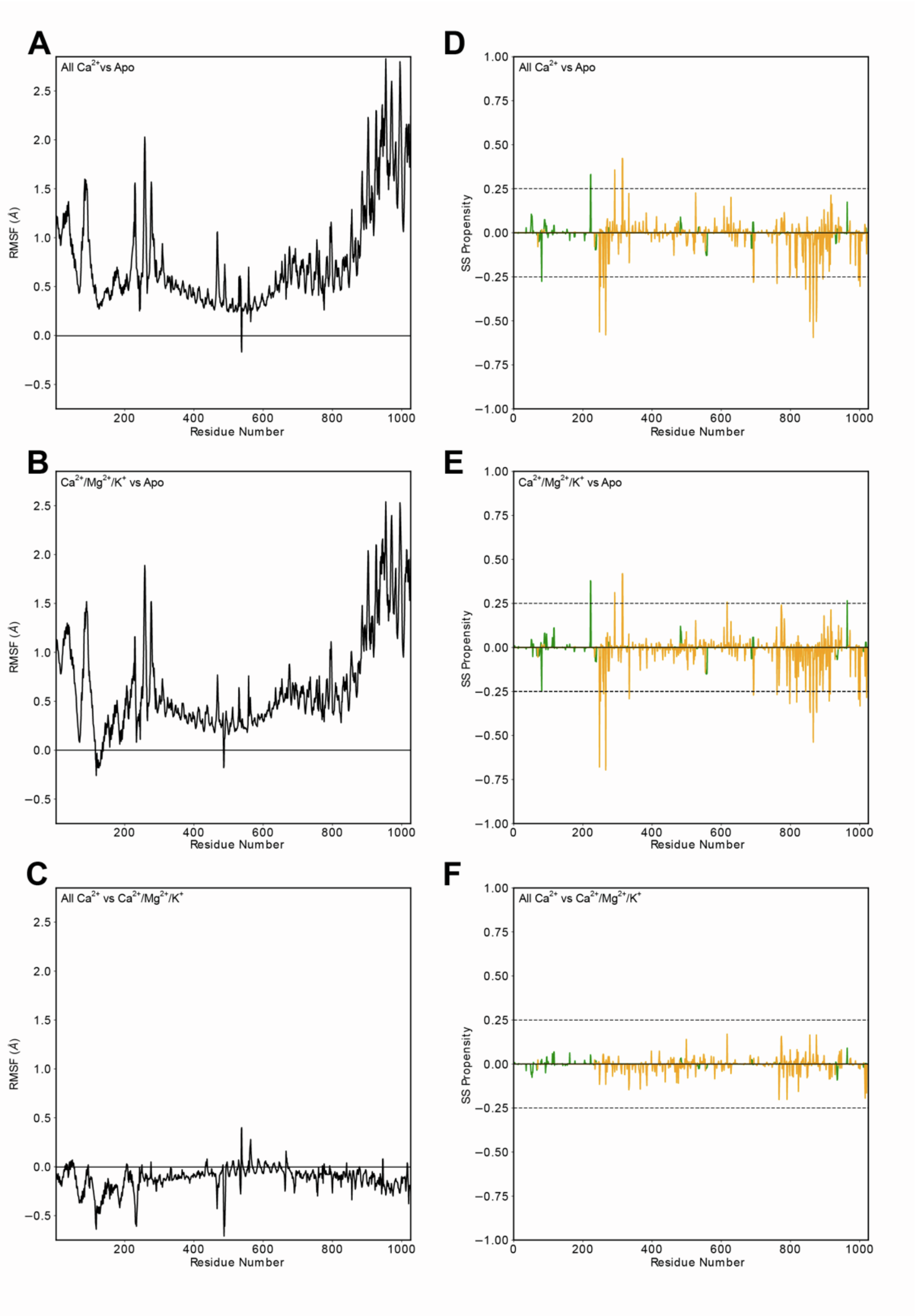

Fig. S3. RMSF and Secondary Structure (SS) propensity in MD simulations (related to Fig. 4).

(A) RMSF differences of the backbone carbons of RsaA, between the all Ca^{2+} simulations and the apo simulation, are plotted along the RsaA sequence. (B) RMSF differences of the backbone carbons of RsaA, between the $Ca^{2+}/Mg^{2+}/K^{+}$ simulations and the apo simulation are plotted along the RsaA sequence. (C) RMSF differences of the backbone carbons of RsaA,

between the all Ca^{2+} and the $Ca^{2+}/Mg^{2+}/K^{+}$ simulations are plotted along the RsaA sequence. (D) SS propensity differences between the all Ca^{2+} simulations and the apo simulation, are plotted along the RsaA sequence. (E) SS propensity differences of the backbone carbons of RsaA, between the $Ca^{2+}/Mg^{2+}/K^{+}$ simulations and the apo simulation are plotted along the RsaA sequence. (F) SS propensity differences of the backbone carbons of RsaA, between the all Ca^{2+} and the $Ca^{2+}/Mg^{2+}/K^{+}$ simulations are plotted along the RsaA sequence. Alpha helical regions are shown in green and beta strands are shown in orange in panels D-F.

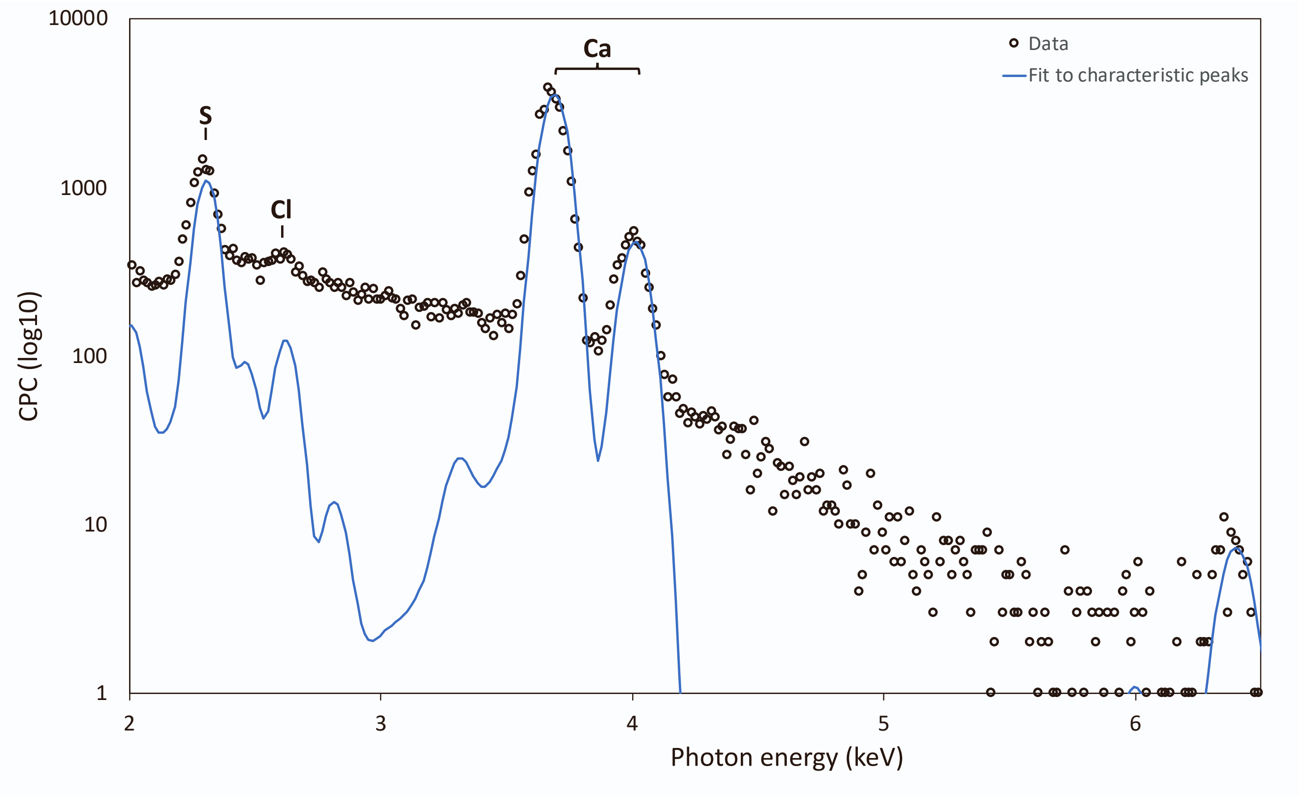

Fig. S4. MicroPIXE analysis of RsaA confirms Calcium binding (related to Fig. 4).

MicroPIXE analysis of full-length, unpolymerized RsaA protein, purified from *C. crescentus* cells shows an average of 8.6 ($\pm$ 0.34) Calcium (Ca) atoms per protein molecule. The *y* axis is displayed as a $\log_{10}$ scale to visualise trace elements. Characteristic peaks for sulphur (S) atoms, chlorine (Cl) and calcium (Ca) respectively are highlighted. Blue line shows the fit to these peaks following background subtraction.

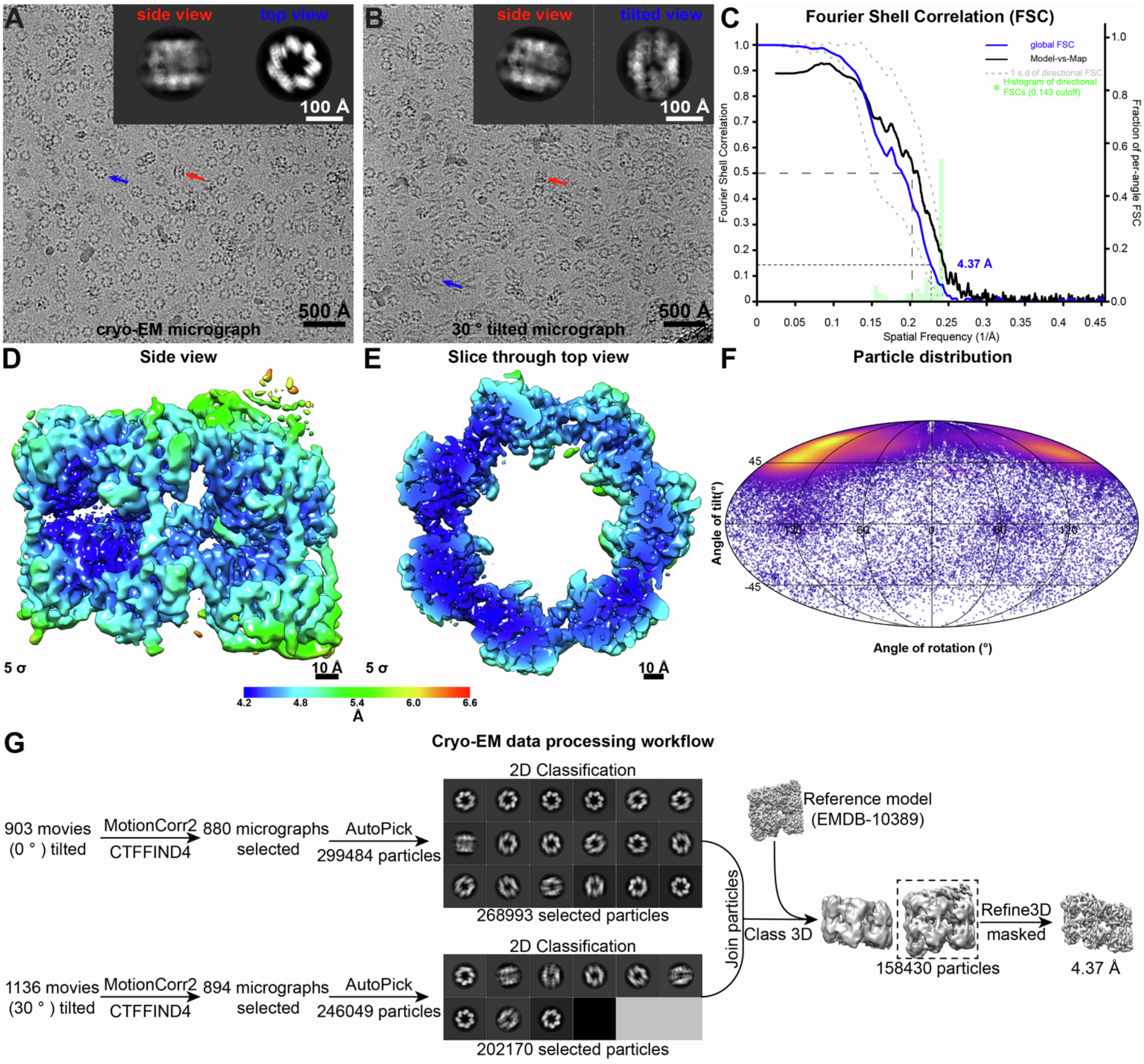

Fig. S5. Cryo-EM single-particle analysis of the Ho^{3+}-bound $RsaA_{NTD}$:PS complex (related to Fig. 5).

(A) Micrographs of the Ho^{3+}-bound $RsaA_{NTD}$:PS complex. Insets shows two-dimensional class averages. (B) Micrographs collected at 30º stage tilt, along with class averages. (C) Resolution estimation of the reconstruction estimated by directional 3D-FSCs (Tan et al., 2017) and model-vs map FSC. (D-E) Local resolution estimation in RELION plotted back onto the cryo-EM map, shown in two orthogonal orientations. (F) Euler angle assignment of particles in refinement. (G) Schematic illustration of the cryo-EM data processing workflow used in this

study. Untilted and tilted dataset were independently pre-processed and merged after 2D-Classification. For 3D-Classification a 30 Å lowpass-filtered reference map (von Kügelgen et al., 2020) (EMDB-10389) of the non-heavy metal replaced specimen was used (see Table S1).

Chain	1	2	3	4	5	6	7	8	9	10	11	12	13	14	15	16	17	18	19
α	Ca	Ca	Ca	Ca	Ca	Ca	Ca	Ca	Ca	Ca	Ca	Ca	Ca	Ca	Ca	Ca	K		Ca
β	Ca	Ca	Ca	Ca	Ca	Ca	Ca	Ca	Ca	Ca	Ca	Ca	Ca	Ca			K		
γ	Ca	Ca	Ca	Ca	Ca	Ca	Ca	Ca	Ca	Ca	Ca	Ca	Ca	Ca	Ca	Ca	K		Ca
δ	Ca	Ca	Ca	Ca	Ca	Ca	Ca	Ca	Ca	Ca	Ca	Ca	Ca	Ca	Ca	Ca			Ca
ε	Ca	Ca	Ca	Ca	Ca	Ca	Ca	Ca	Ca	Ca	Ca	Ca	Ca	Ca		Ca	K		
ζ	Ca	Ca	Ca	Ca	Ca	Ca	Ca	Ca	Ca	Ca	Ca	Ca	Ca	Ca	Ca	Ca	K*		Ca

** A density is visible but it is shifted away from the canonical metal-binding site.*

Fig. S6. Long-wavelength anomalous X-ray diffraction experiments reveal extensive Ca^{2+} binding in $RsaA_{CTD}$ (related to Fig. 6).

Tabular summary of long-wavelength anomalous X-ray diffraction experiments conducted on three-dimensional crystals of $RsaA_{CTD}$. Six monomers of RsaA within the $P2_1$ asymmetric unit of RsaA are labelled α- ζ. Original proposed Ca^{2+} binding sites 1-19 are labelled with their observed densities that include Ca^{2+} (green), K^+ (red), or no occupancy (grey), at a contour level of 4.5 σ. For position 17 in ζ, the K+ density is heavily shifted away from the proposed metal-ion site, and in general, the shapes of the anomalous density for K^+ also suggest that the ions are not bound very tightly, in line with the MD simulations.

Supplementary Tables

Table S1: Cryo-EM data collection, refinement and validation statistics **(related to Fig. 5).**

#RsaA_NTD Ho^{3+} soak<br>(EMDB-13355)<br>(PDB 7PEO)<br>(EMPIAR-10790)		
**Data collection and processing**		
Microscope	Titan Krios G3	Titan Krios G3
Magnification	130,000	130,000
Voltage (kV)	300	300
Electron exposure ($e^{-}/Å^2$)	44.8	44.8
Stage Tilt (°)	0	30
Detector	Gatan K2 SUMMIT	Gatan K2 SUMMIT
Slit width (eV)	20	20
Defocus range (μm)	-1 to -3	- 1 to -3
Pixel size (Å)	1.08	1.08
Symmetry imposed	C1	C1
Micrographs collected (no.)	903	1,115
Micrographs used (no.)	880	894
**Data processing**		
Software	RELION3.0*	RELION3.0*
Initial particle images (no.)	299,484	246,049
Final particle images (no.)	158,430	
Rescaled Box-size Class2D (px)	150 x 150	
Initial 3D reference map used (EMDB code)	10,389	
Box-size Class3D (px)	300 x 300 x 300	
Final Box-size (px)	300 x 300 x 300	
Pixel size final reconstruction (Å)	1.08	
Symmetry	C1	
Map resolution (Å)	4.37	
FSC threshold	0.143	
Map resolution range (Å)	4.2-6.6	
Map sharpening *B* factor ($Å^2$)	-181.397	
3D FSC sphericity#	0.888	
Map CC (mask)	0.87	
Map CC (volume)	0.86	

Map CC (peaks)		0.79
**Model Refinement**		
Initial model used (PDB code)	6T72	
Software	Refmac5	
Algorithm	Reciprocal	
Model resolution (Å)	4.5	
FSC threshold	0.5	
Model composition		
Non-hydrogen atoms	26278	
Protein residues	3388	
Ligand residues	168	
Ions	42	
B factors ($Å^2$)		
Protein	249.69	
Ligand	296.65	
R.M.S. deviations		
Bond lengths (Å)	0.008	
Bond angles (°)	1.465	
Validation		
MolProbity score	1.25	
Clashscore	3.60	
Poor rotamers (%)	0.60	
Cβ outliers (%)	0.00	
CABLAM outliers (%)	1.68	
Ramachandran plot		
Favored (%)	97.50	
Allowed (%)	2.50	
Outliers (%)	0.00	

[*] Cryo-EM reconstruction (Zivanov et al., 2018).

[#] 3DFSC sphericity as determined by the methods described in (Tan et al., 2017).

Table S2. Crystallographic data for $RsaA_{CTD}$ protein crystals used in long-wavelength X-ray diffraction experiments* (related to Fig. 6).

	**Crystal 1**		**Crystal 2**	
E [keV]	4.1	3.95	3.7	3.55
Wavelength [Å]	3.024	3.139	3.351	3.492
Space group	$P2_1$	$P2_1$	$P2_1$	$P2_1$
Unit cell [Å,°]	215.70, 73.84, 222.71, 90.000, 118.644, 90.000	216.43, 74.11, 222.93, 90.000, 118.606, 90.000	215.47, 76.65, 222.33, 90.000, 118.793, 90.000	216.17, 76.68, 223.00, 90.000, 118.835, 90.000
Resolution range [Å]	196 – 3.70 (3.80-3.70)	196-4.05 (4.16-4.05)	195-3.80 (3.90-3.80)	195-4.25 (4.36-4.25)
Total reflections	410145 (26569)	318946 (20923)	361849 (16115)	280109 (17635)
Unique reflections	121604 (8145)	94660 (6656)	110514 (5269)	83564 (5605)
Multiplicity	3.4 (3.3)	3.4 (3.1)	3.4 (3.1)	3.4 (3.1)
Completeness [%]	94.3 (85.7)	95.4 (90.2)	89.8 (58.1)	94.4 (86.1)
Mean $I/\sigma(I)$	4.17 (0.88)	4.33 (0.85)	3.88 (0.93)	3.79 (0.95)
$CC_{1/2}$ (%)	98.4 (62.8)	98.6 (53.8)	98.8 (57.2)	99.0 (49.2)
Wilson B [$Å^2$]	58.7	65.7	68.9	62.9
R_{merge} [%]	30.6 (142.9)	29.9 (173.6)	28.5 (147.1)	29.3 (146.7)
R_{meas} [%]	36.4 (170.8)	35.6 (208.7)	34.1 (176.7)	34.9 (176.6)

* Raw diffraction data of these experiments have been deposited at http://www.proteindiffraction.org with the DOI - https://doi.org/10.18430/M3.IRRMC.5999

Legends for Movies S1 and S2

Movie S1. Cryo-EM map of the Ho^{3+}-bound $RsaA_{NTD}$:PS complex (related to Fig. 5).
This movie shows different views of the Ho^{3+}-bound $RsaA_{NTD}$:PS complex. Due to strong densities corresponding to bound Ho^{3+} ions, the map is contoured at 18 σ away from the mean (black mesh), overlaid on the fitted atomic structure of $RsaA_{NTD}$ and the bound PS.

Movie S2. Long wavelength X-ray diffraction experiments on $RsaA_{CTD}$ crystals (related to Fig. 6).
Anomalous difference maps showing positions of Ca^{2+} ions (green mesh, 4.5 σ) and K^{+} ions (magenta mesh, 4.5 σ) are overlaid on the $RsaA_{CTD}$ atomic structure, as shown in Fig. 6.
